# Multiplex immunophenotyping of human acute myeloid leukemia patients revealed single -cell heterogeneity with special attention on therapy sensitive and therapy resistant subpopulations

**DOI:** 10.3389/fimmu.2025.1563386

**Published:** 2025-04-17

**Authors:** Nikolett Gémes, Benedek Rónaszéki, Szabolcs Modok, Zita Borbényi, Imre Földesi, Éva Trucza, Blanka Godza, Zsuzsanna László, Balázs Csernus, László Krenács, Enikő Bagdi, Enikő Szabó, László G. Puskás, Valeria Bertagnolo, Gábor J. Szebeni

**Affiliations:** ^1^ Laboratory of Functional Genomics, Core Facility, HUN-REN Biological Research Center, Szeged, Hungary; ^2^ Department of Internal Medicine, Hematology Center, Faculty of Medicine, University of Szeged, Szeged, Hungary; ^3^ Faculty of Medicine, Institute of Laboratory Medicine, University of Szeged, Szeged, Hungary; ^4^ Department of Medical Genetics, University of Szeged, Szeged, Hungary; ^5^ 1st Department of Pathology and Experimental Cancer Research, Faculty of Medicine, Semmelweis University, Budapest, Hungary; ^6^ Laboratory of Tumor Pathology and Molecular Diagnostics, Szeged, Hungary; ^7^ Avidin Ltd., Szeged, Hungary; ^8^ University of Ferrara, Department of Morphology, Surgery and Experimental Medicine, Ferrara, Italy; ^9^ Astridbio Technologies Ltd., Szeged, Hungary

**Keywords:** single-cell immunophenotyping, leukemia-associated immunophenotype, acute myeloid leukemia, drug resistance, minimal residual disease, luminex MAGPIX

## Abstract

**Introduction:**

Understanding leukemia-associated immunophenotypes (LAIP) could assist in the design of therapies to ameliorate patient benefits in acute myeloid leukemia (AML). In our study, focusing on single-cell heterogeneity in therapeutic resistance, flow cytometric immunophenotyping of the peripheral blood of therapy-naive and follow-up AML patients versus age and sex-matched healthy controls (HCs) was performed.

**Methods:**

The FACS panel consisted of Viobility 405/520 Fixable Dye, Anti-human CD45, CD19, CD3, CD7, CD33, CD34, CD38, CD64, CD117, CD135, HLA-DR antibodies. Unsupervised clustering algorithms such as Uniform Manifold Approximation and Projection for Dimension Reduction (UMAP) and Flow cytometry data that builds Self-Organizing Maps (FlowSOM) were used to reveal the LAIP. The measurable residual disease (MRD) was monitored by our proposed manual gating. To complement the characterization of peripheral immune cells, Luminex MAGPIX was used to measure the concentration of 31 soluble immune-oncology mediators from the plasma of AML patients and HC.

**Results:**

Both manual gating, UMAP and FlowSOM showed normalization of LAIP similar to the HC immune landscape following therapy. Eleven metaclusters (MCs) were associated with AML before therapy. The follow-up of AML samples revealed four MCs of therapy sensitive cells, and one MC composed of therapeutic resistant cells (MC12: CD3-CD7-CD33-CD38- CD64- HLA-DR- CD117- CD135-) identified by the FlowSOM analysis. The initial AML blasts in the MRD gate (CD19-, CD45+, CD3-, CD38+/CD34±, CD7+/CD117+, CD117+/CD135+) were detectable at the lowest frequency in our current study at 22 cells per 100,000 (0.022%) CD45+CD3- living singlet parental population. In the plasma of AML patients the levels of BAFF, B7-H2, B7-H4, CD25, MICA, and Siglec-7 were increased versus HCs.

**Conclusions:**

This study focused on understanding the LAIP in AML before and after therapeutic intervention. The study highlights the potential of using single-cell LAIP profiling and immune mediator measurements to monitor therapy response and identify measurable residual disease and therapy resistant cell populations in AML.

## 1 Introduction

Acute myeloid leukemia (AML) is a biologically complex and clinically heterogeneous disease, that remains a big challenge for physicians. The estimated 5-year overall survival (OS) of this bone marrow stem cell cancer is only 30% and despite of novel therapies, there is still no breakthrough to remarkably improve the outcome (1). AML is the most common form of acute leukemia in adults, which is typically diagnosed in the late 60’s (2). Peripheral blood and bone marrow examination (immunohistochemistry IHC, molecular analysis, immunophenotyping, and genetics) are essential for establishing an accurate diagnosis based on the latest WHO guideline (2022 WHO criteria), International Consensus Classification (2022 ICC), and European LeukemiaNet (2022 ELN) (3). Clinicians face many challenges in the management of AML. First, at the diagnosis time, physicians must determine who is fit or unfit (“frail”) for standard treatment among elder adults which is always a difficult task. The enumeration of adequate performance status and comorbidity burden could be helpful, but these parameters are still not sufficient to make reassuring decisions (4). Second, AML is a biologically heterogeneous disease; therefore, the identification and follow-up of the leukemia-associated immunophenotype (LAIP) or cells that are different-from-normal (DfN) is not an easy task. Therefore, it is essential to detect and describe the characteristics of neoplastic cells at the time of diagnosis (5, 6). Third, minimal or measurable residual disease (MRD) measurement is an emerging prognostic factor that could be helpful in decision-making for AML management. MRD status could be a game changer in the future of allogeneic stem cell transplantation (allo-SCT) recommendations in AML, which could be significant from the perspective of therapy-related toxicity, and transplant-related mortality, and to lower the allocational and financial burden of allo-SCT (7, 8).

There are multiple options which are available for MRD follow-up, methods like multicolor flow cytometry (MFC) by the identification of highly specific leukemia-associated immunophenotypes (LAIPs) or specific genetic mutation measurement by real-time quantitative PCR (qPCR), digital droplet PCR or by next-generation sequencing (NGS) (5, 9), but these methodologies are unavailable for every hematology center, and hematologists are still waiting for better access to the daily routine. The lack of standardized protocols is another problem in MRD measurement (10). We focused in the current work on the measurement of the LAIP with multi-dimensional data mining of single cells with attention to the determination of therapy-sensitive or resistant AML subpopulations by FlowSOM, and we showed the detection of MRD by manual gating also. The panel design was based on literature data, cluster of differentiation (CD) markers incorporated in our single-center study were the following: CD45 a protein tyrosine phosphatase exclusively expressed on all nucleated cells of the hematopoietic system, we used CD19 to exclude B-cells from the analysis (11), CD3 is a part of the T-cell- and pre-T-cell receptors (12), CD7 is expressed by the leukemic blasts and malignant progenitor cells of approximately 30% in AML patients, but it is absent on the surface of normal myeloid and erythroid cells. CD33 molecule is expressed in 80–90% of all AML (13). CD34 is expressed by immature hematopoietic cells such as myeloid and lymphoid progenitors, erythroid and multipotential progenitors, and lymphohematopoietic stem cells (14). AML blasts tend to express CD34 as well (15), but CD34 negative AML is also known (16). CD38 is a multifunctional extracellular enzyme on the cell surface with nicotinamide adenine dinucleotide nucleosidase and cyclase activities (17). CD64 is a transmembrane protein with broad expression on the surface of various types of AML cells, especially monocytic AML cells, but it is absent on the surface of hematopoietic stem cells and most of non-monocytes (18). In AML, CD117 is an important diagnostic marker, and could also be a prognostic factor in some subtypes of AML (19). CD135 also known as the FLT3 protein, it is expressed almost exclusively in the hematopoietic compartment (20). CD135 is a prognostic factor, a new marker for minimal residual disease, and a potential novel therapeutic target for AML (21). HLA-DR was also measured, because its low level might compromise CD4+ T-cell-mediated anti-tumor immunity in AML (22).

The AML subtypes incorporated in our single-center study highlighted by the characteristic immunophenotype are as follows: In acute myeloblastic leukemia, with minimal myeloid differentiation (former AML-M0, FAB = French-American-British classification) blasts originate from the early stage of myeloid progenitors. From the perspective of immunophenotype blasts are MPO and CD34 positive with CD117 co-expression (23). The acute myeloblastic leukemia with maturation (former AML-M2 FAB) is often positive for CD13, CD15, CD33, CD34, CD117, HLA-DR and comprises approximately 25-30% of all cases with ≥ 20% blasts in the bone marrow or peripheral blood with ≥ 10% granulocyte differentiation (24). In acute myelomonocytic leukemia (former AML-M4, FAB); AMMoL; and AMML), blasts show monocytic and granulocytic features. It represents 5 - 10% of all cases of AML. From the perspective of immunomorphology blasts express two or more myeloid-associated antigens, such as MPO, CD13, CD33 and CD117 (25). The acute monocytic leukemia (former AML-M5, FAB), or alternatively acute monoblastic and acute monocytic leukemia represents 10% of AML cases (23). This type is characterized by the expression of at least two monocytic markers from the following list: CD4, CD14, CD11b, CD11c (50%), CD36, CD64, CD68, and HLA-DR. The following myeloid markers are also expressed by blast-like cells: CD13, CD33, CD15, CD65, CD34, CD117, and MPO (26, 27).

In our single-center study, we aimed to collect multiple subtypes of non-APL AML patients as therapy-naive cases and follow-up samples after treatment to define their immunophenotype using MFC. Additionally, the MRD cells were followed by our 12-plex MFC panel, the same as used for the immunophenotyping of therapy naive AML blasts. To complement the immunophenotyping of peripheral AML cells, the concentrations of 31 soluble immune-oncology mediators were measured. In this paper, we propose the application of unsupervised UMAP data visualization tool that reduces dimensions in a two-dimensional space and FlowSOM analysis for deciphering the LAIP in AML.

## 2 Materials and methods

### 2.1 Ethical statement and study design

Studies involving humans were approved by the Ethics Committee of the National Public Health Center, Hungary under the 60440-6/2021/EÜIG Project identification code. The study was conducted in accordance with the Declaration of Helsinki and was in agreement with local legislation and institutional requirements. All participants provided their written informed consent to participate in the study.

The study involved AML patients (n=14, median age: 54.3 years, 35.7% male) and healthy controls (HCs, n=14, median age: 55.5 years, 35.7% male), who were matched by age and sex. The demographic data for the HCs can be found in **Supplementary Table 2**. Patient enrollment: We included newly diagnosed, treatment-naive AML patients aged 18 years or elder. All participants were managed by the Hematology Centre at the Department of Internal Medicine, Szent-Györgyi Albert Medical Centre, Szeged. Individuals who had previously received antileukemia treatment were excluded from the study.

### 2.2 Sample preparation for flow cytometry

Cell isolation was performed as previously described by our group (28). Briefly, 10 ml of peripheral blood was collected into Lithium Heparin tubes (BD Vacutainer, Beckton Dickinson). Peripheral blood mononuclear cells (PBMCs) were isolated by gradient centrifugation using Leucosep tubes (Greiner Bio-One) according to the manufacturer’s instructions. PBMCs (5x10^6^/ml) were stored in liquid nitrogen in freezing media (90% FBS, Capricorn, 10% DMSO, Merck, v/v%). Additionally, 200 µl aliquots of plasma were stored at -80°C for the Luminex MAGPIX assay.

### 2.3 Flow cytometry

Flow cytometry was performed as previously described by our group with minor modifications (28, 29). PBMCs were thawed in a 37°C water bath, washed with 10 ml RPMI medium (Capricorn), and centrifuged at 360 x g for 5 minutes. The cells were suspended in 5 ml RPMI and counted in a Bürker chamber using trypan blue exclusion viability dye (Merck). A total of 0.5 x 10^6^ cells were stained for flow cytometry. Viobility dye 405/520 nm (Miltenyi Biotec) was added to the cells according to the manufacturer’s instructions (100X dilution), with 0.5 µl added to 49.5 µl PBS. The cells were incubated at room temperature in the dark for 15 min., then washed with 1 ml immune fluorescence buffer (IFB; 2% FBS in PBS, v/v%). After centrifugation at 360 x g for 5 min., cells were incubated with 5 µl TrueStain Fc receptor blocking (BioLegend) solution in 95 µl IFB for 10 min. 500 µl IFB was added for washing. Ater centrifugation at 360 x g for 5 minutes the supernatant was discarded, an antibody cocktail was prepared in IFB to a final volume of 100 µl per sample, containing 2.5 µl each of anti-CD135, anti-CD34, anti-CD64, and 1.25 µl each of anti-CD45, anti-CD19, anti-CD3, anti-CD7, anti-CD33, anti-CD38, and anti-CD117 (BioLegend). The antibodies used for MFC are listed in **Table 1**. The cells were incubated with the antibody cocktail at room temperature in the dark for 45 min., then washed with 1 ml IFB and centrifuged at 360 x g for 5 minutes. The cells were suspended in 350 µl IFB and acquired on a Cytoflex S flow cytometer (Beckman Coulter). The gating strategy in CytExpert v2.4.0.28 (Beckman Coulter) is shown in **Supplementary Figure 14**. UMAP visualization and FlowSOM analyses were performed using FlowJo v10.10.0 (Becton Dickinson) (30, 31).

**Table 1:** The list of the dyes/antibodies used for MFC.

Dyes/Antibodies (Clone)-Fluorochrome	Vendor, Cat. number
Viobility 405/520 Fixable Dye	Miltenyi Biotec, 130-109-814
Anti-Human CD45 (2D1)-Pacific blue	Biolegend, 368540
Anti-Human CD19 (HIB19)-Brilliant Violet 510	Biolegend, 302242
Anti-Human CD3 (OKT3)-Brilliant Violet 605	Biolegend, 317322
Anti-Human CD7 (CD7-6B7)-PE/Dazzle™ 594	Biolegend, 343120
Anti-Human CD33 (WM53)-Brilliant Violet 650	Biolegend, 303430
Anti-Human CD34 (561)-Alexa Fluor^®^ 488	Biolegend, 343620
Anti-Human CD38 (HB7)-APC	Biolegend, 356606
Anti-Human CD64 (10.1)-Alexa Fluor^®^ 700	Biolegend, 305040
Anti-Human CD117 (104D2)-APC/Cyanine7	Biolegend, 313228
Anti-Human CD135 (BV10A4H2)-PE/Cyanine7	Biolegend, 313314
Anti-Human HLA-DR (LN3)-PerCP/Cyanine5.5	Biolegend, 327020

### 2.4 Statistics

Statistical analysis was performed using Graphpad Prism Version 8.4.2 (Dotmatics). The normal distribution and lognormality of the data sets were tested using the Shapiro-Wilk, D’Agostino & Pearson, and Kolmogorov-Smirnov normality and lognormality tests. The assay specific statistical analyses are indicated in each figure legend.

#### 2.4.1 Statistics of flow cytometry data

FlowSOM I: Statistical analysis was performed on 14 HCs and 14 patients with AML. To compare healthy controls and patients with AML non-parametric Mann-Whitney unpaired rank test was used.

FlowSOM II: Statistical analysis was performed with six AML follow-up patients and six healthy controls. Normally distributed datasets were compared with parametric RM (repeated-measures) one-way ANOVA paired test, and the Friedman test was applied for non-parametric analysis.

All types of statistical tests were corrected for multiple comparison by controlling the False Discovery Rate (FDR) using two-stage Benjamini, Krieger and Yekutieli approach with an FDR cutoff of 10%. The differences were considered significant at *p < 0.05; **< 0.01; ***< 0.001. The GraphPad Prism diagrams are box and whisker plots, set to show ‘min to max, show all points’.

## 3 Results

### 3.1 Immunophenotyping of human therapy-naive AML cases versus healthy controls using multiplex flow cytometry and dimensional reduction analysis of single-cell data

**Figure 1:**
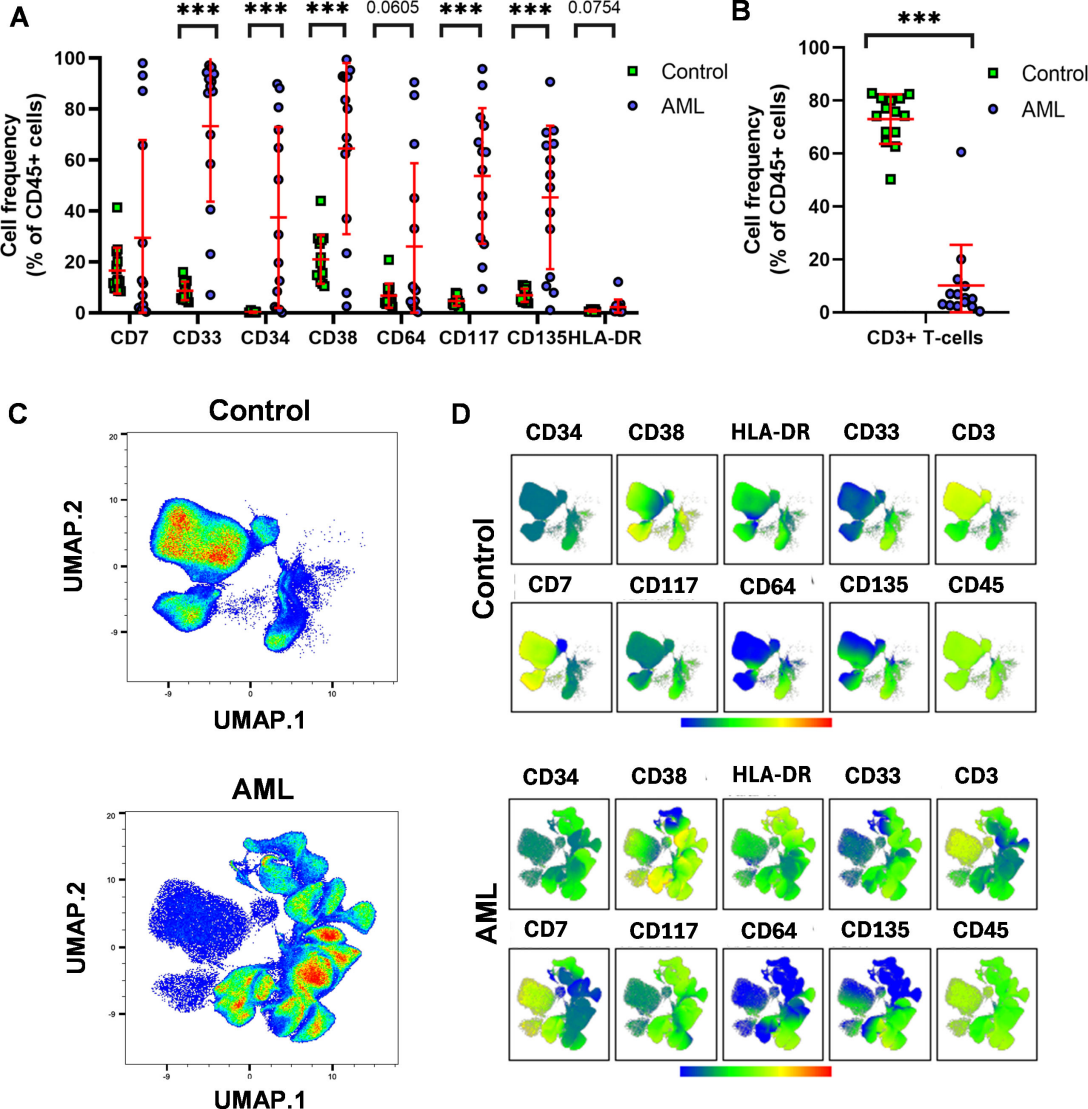
The leukemia-associated immunophenotype (LAIP) of therapy-naive AML. **(A)** Flow cytometry was assessed to measure the cell frequency within the CD45+CD3- living singlets (red lines: mean ± SD). **(B)** T-cell deficiency of the AML patients under evaluation by manual gating. **(C)** The Uniform Manifold Approximation and Projection (UMAP) visualization of leukocyte distribution in AML vs. HCs. The UMAP was launched on live CD19- single cells(manually gated in FlowJo), cell number for UMAP: 22800/case; data were generated from 638,400 single cells from 24 cases (n=14 HC, n=14 AML). The coloration is proportional with the cell density from low (blue) to high (red). **(D)** The marker expression intensities of UMAP visualized cell clusters in AML vs. HCs. The coloration is proportional with the marker expression intensity from low (blue) to high (red). The **(C)** and **(D)** represent aggregated (concatenated) data of the n=14 AML and n=14 HC samples. The differences were considered significant at ***< 0.001.

Flow cytometry was performed by manual gating for immunophenotyping on CD45+CD3- cells using the AML antibody panel designed in our laboratory based on literature data (**Figure 1A**). Five of the investigated markers showed significantly higher expression and discrimination between AML samples and age- and sex-matched healthy controls (HCs). Namely, the percentages of cells positive for a given marker in the AML versus (vs.) HC samples were as follows: (mean of the 14 cases): CD33 (73.2 vs. 8.5%), CD34 (37.4 vs. 0.2%), CD38 (68.5 vs. 20.9%), CD117 (53.7 vs. 4.6%), CD135 (45.3 vs. 6.8%) (**Figure 1A**). The low T-cell count was obvious in the therapy-naive AML samples compared to HCs (10.1 vs. 72.9%) (**Figure 1B**). The gating strategy for manual gating is shown in **Supplementary Figure 14**. Next, we aimed to demonstrate the power of the Uniform Manifold Approximation and Projection (UMAP) for visualization of leukocyte distribution in AML vs. HCs. The UMAP plots placed the cells in a multi-dimensional mathematical space to create an immune landscape in which the determinants are the expression of the 10 investigated markers. In **Figure 1C**, the UMAP projection is grouped by sample types (therapy-naive AML and HCs) and the color of UMAP indicates the cell density of the sub-groups. UMAP showed an AML-characteristic cell distribution with high inter-cellular heterogeneity within the AML group (higher segmentation of the identified subpopulations), while HCs only formed a small number of clusters. Additionally, the marker expression intensities of the 10 investigated proteins were demonstrated on the UMAP plots to delineate sub-populations of AML samples vs. HCs (**Figure 1D**).

**Figure 2:**
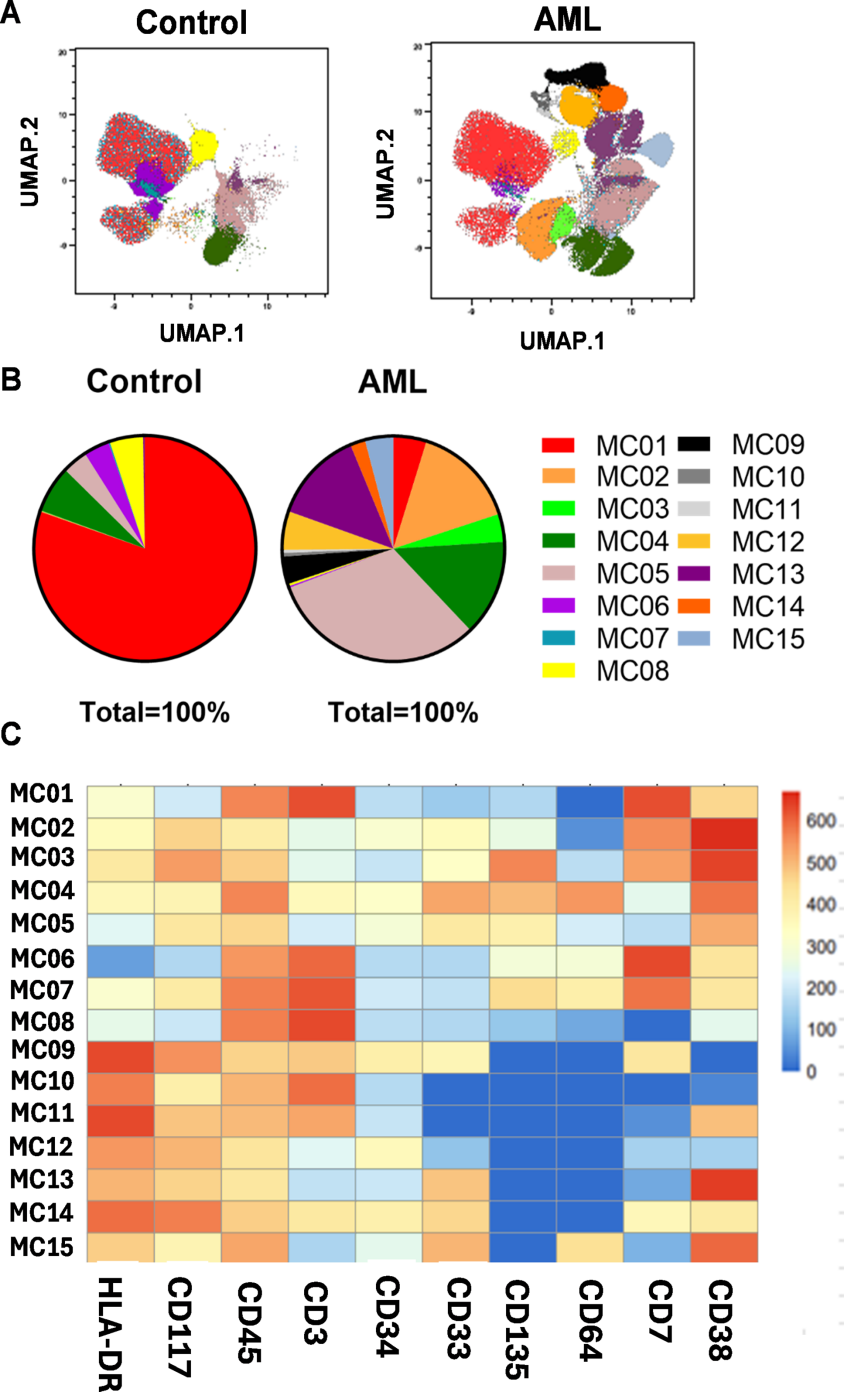
Identification of AML subpopulations (metaclusters = MC) based on single-cell flow cytometry data. **(A)** The FlowSOM (Flow cytometry data that builds Self-Organizing Maps) analysis together with UMAP visualization shows the arrangement of the MCs01-15 in the 10-dimensional mathematical space in AML vs. HCs. The colors from 01-15 label the identified 15 MCs. **(B)** The pie chart shows the frequencies of MC distribution in AML vs. HCs. **(A, B)** Concatenated data of the investigated AML (n=14) or HC (n=14) samples. **(C)** The heatmap demonstrates the marker expression intensity of the identified MCs. The coloration is proportional with the extent of marker expression for blue (low expression) to red (high expression). The heatmap shows concatenated data for the 15 MCs of the 28 cases analyzed.

To better determine the AML related sub-populations, FlowSOM analysis (Flow cytometry data that builds Self-Organizing Maps) was performed following UMAP. Fifteen metaclusters (MCs01-15) were identified as subgroups of the analyzed cases (**Figures 2A, B**). The population frequencies of MCs01-15 of AML patients vs. HCs are shown in **Supplementary Figure 15**. The immunophenotype of healthy controls was composed of mainly six MCs: MC01 (80.3%), MC04 (6.7%), MC05 (3.6%), MC06 (3.7%), MC07 (0.19%), MC08 (4.8%) (**Figure 2B**), the MC01 (HLA-DR+, CD117-, CD45+, CD3+, CD34-, CD33-, CD135-, CD64-, CD7+, CD38+); MC06 (HLA-DR-, CD117-, CD45+, CD3+, CD34-, CD33-, CD135low, CD64low, CD7+, CD38+); MC07 (HLA-DRlow, CD117+, CD45+, CD3+, CD34-, CD33-, CD135+, CD64+, CD7+, CD38+), MC08 (HLA-DR-, CD117-, CD45+, CD3+, CD34-, CD33-, CD135-, CD64-, CD7-, CD38low) MCs discriminated HCs significantly against AML (**Supplementary Figure 15**). The immunophenotype of AML composed of mainly MC02 (15.1%), MC03 (4.0%), MC04 (13.8%), MC05 (31.5%), MC09 (3.9%), MC10 (0.51%), MC11 (0.43%), MC12 (5.5%), MC13 (13.2%), MC14 (2.1%), MC15 (4.1%) (**Figure 2B**). Exclusively, the presence of the following MCs discriminated therapy -naive AML from HCs: MC02, MC03, MC09, MC10, MC11, MC12, MC13, MC14, and MC15, however, these were not statistically significant. The heatmap of marker expression intensities facilitates the understanding of the MC subgroups (**Figure 2C**). The largest AML-related MC was MC05, with the following immunophenotype: HLA-DR-/CD117+/CD45+/CD3-/CD34low/CD33+/CD135+/CD64-/CD7-/CD38+ (**Figure 2C**).

**Figure 3:**
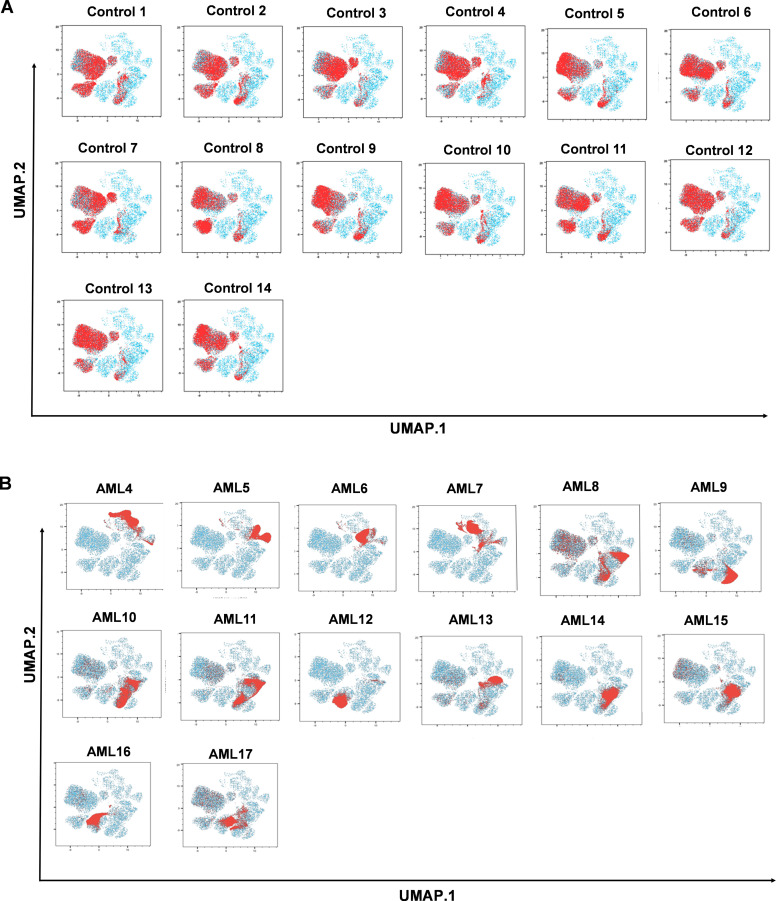
The immune landscape of the individual AML or HC subjects based on UMAP visualization. The red clouds represent the cells of the **(A)** healthy controls (n=14), and **(B)** AML cases (n=14) under investigation in the 10-dimensional mathematical space created by the UMAP visualization tool (blue color).

The previously showed results helped to discriminate therapy-naive AML from the HC group and demonstrated the power of UMAP and FlowSOM in deciphering the single-cell heterogeneity and subpopulation frequency of AML. Here, we highlight an analysis of individual cases that may assist personalized medicine-based decisions in the future. Plotting the HC subjects (n=14) separately on the cellular distribution map showed that the enrolled HCs were completely homogenous in terms of the immunophenotype (**Figure 3A**). In contrast, investigation of AML cases separately by single-cell resolution showed different immunophenotypes in individual patients (**Figure 3B**). There was no complete overlap between two AML patient groups in the profile. For complete characterization of patients, the historical FAB, IHC, FACS, and FISH (fluorescence *in situ* hybridization) diagnostic data are summarized in **Supplementary Table 3**. The individual MC distributions for each subject are shown in **Supplementary Figure 16**. However, the following patient categories can be determined based on similar MC compositions: (1) AML8, AML9, AML10, and AML11. (2) Another patient subgroup was AML13, AML14, and AML15, with similar MC composition. (3) The third patient subgroup was created from AML12 and AML16 (**Supplementary Figure 16**). The remaining samples, namely AML4, AML5, AML6, AML7, and AML17, exhibited unique expression patterns for the 10 proteins studied.

### 3.2 The follow-up of the LAIP after therapeutic response

**Figure 4:**
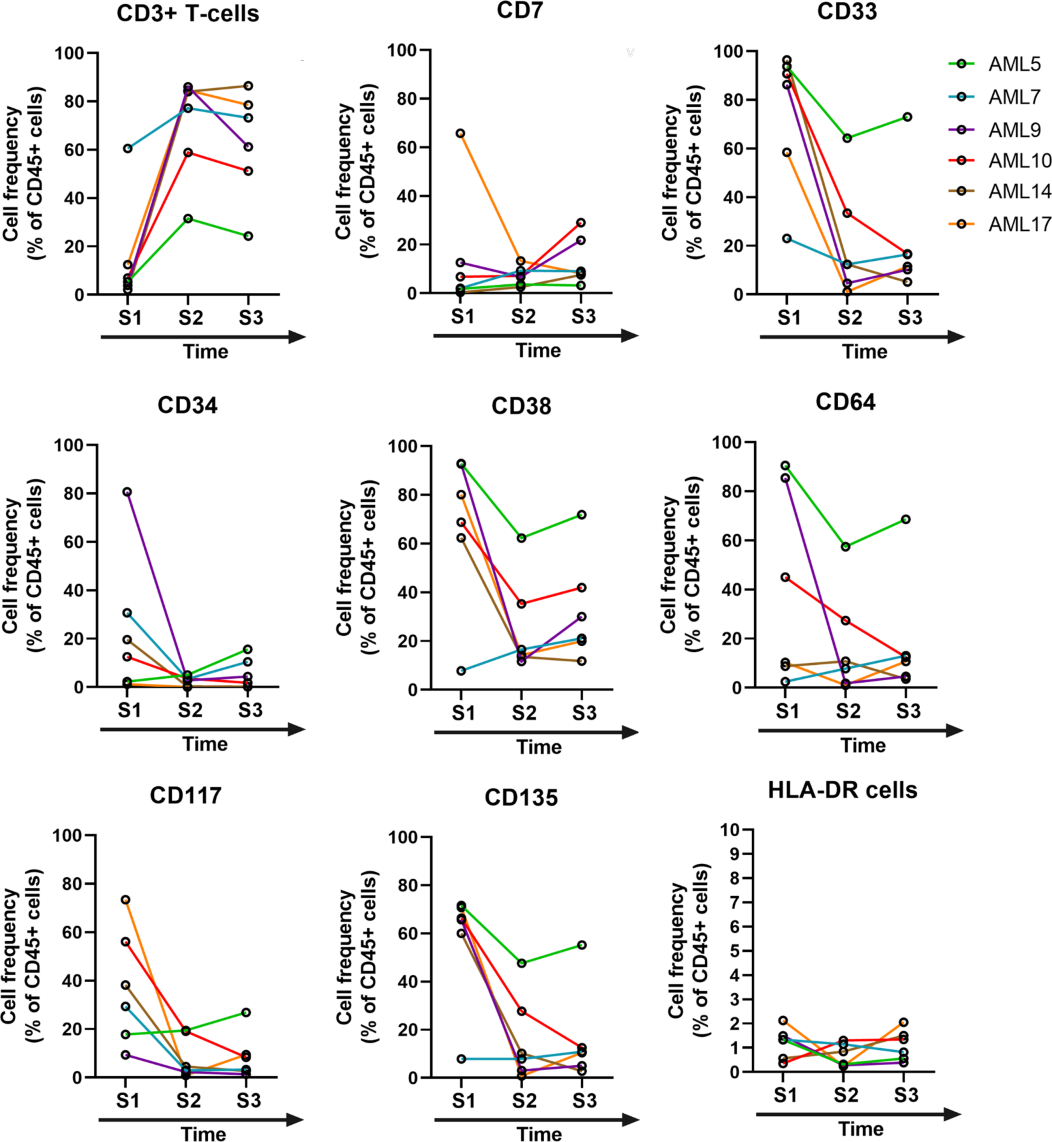
The time course of post-treatment changes in the marker expression profile. Manual gating was used in flow cytometry to determine the cell frequency within the CD19– living, and CD45+ single-cell population of the AML cases (n=6). After gating the CD3+ T-cells, the rest of the markers were gated on the CD45+CD3- compartment. S1= sample 1, S2= sample 2, S3= sample 3.

After obtaining results about the single-cell heterogeneity of therapy-naive AML patients, our second aim was to decipher the changes in LAIP following therapy. In the first instance, manual gating was used to measure alterations in cell frequencies following therapeutic interventions (S2 and S3) within the CD19- living, CD45+ population (**Figure 4**). The before-after plots demonstrate the restoration of CD3+ T-cell number, and in separate analysis the follow-up of CD7+, CD33+, CD34+, CD38+, CD64+, CD117+, CD135+, and HLA-DR+ cells within the CD19- living, CD45+CD3- gate is shown. Unfortunately, eight patients died following induction therapy; therefore, follow-up immunophenotyping was performed on six subjects. In some cases, complete remission was observed, such as decreased expression of CD33, CD38, and CD135 (AML9, AML10, AML14, AML17), and diminished levels of CD117 (AML7, AML9, AML10, AML14, AML17) (**Figure 4**).

Manual gating identified the MRD population. First, the cells, singlets, CD19- viable cells, CD45+/CD3- cells were gated as parental cells as shown in **Supplementary Figure 14A**. Then CD38+/CD34+ and CD38+/CD34- cells were gated in one gate (P1), subsequently CD117+/CD7+ (P2) were defined; then CD117+/CD135+ cells were gated (P3) (**Supplementary Figure 17**). The initial AML blasts in the MRD gate were detectable at the lowest frequency in our current study at 22 cells per 100,000 (0.022%) CD45+CD3- living singlet parental population (AML14) at time zero (S1). The sensitivity of detection was 10^-4^ (0.00022 MRD cell/1 CD45+CD3- living singlet). During the follow-up period the cells in the MRD gate decreased from time zero to the third sampling in the case of AML9 from 1199 to 299 cells (0.29%), for AML10 from 2830 to 468 cells (0.46%), for AML17 from 53739 to 642 cells (0.64%) per 100,000 CD45+CD3- living singlet parental population. AML9, AML10, and AML17 represent longer survival with 860, 846, and 412 days at the time of publication, those individuals are still alive. Interestingly, the MRD population increased in the case of AML14 from 22 to 820 cells (0.82%), but this subject with MRD under 1% is also showed longer survival of 561 days and still alive (**Supplementary Figures 17B, C**). Unfortunately, AML5 passed away due to an infection, which was related to the preparation for BM transplantation, and AML7 deceased due to long-term leukopenia. The MRD of AML5 increased from 327 to 499 (0.49%); in the case of AML7, the MRD increased from 123 to 320 cells (0.32%). The overall survival (OS) of patients with AML is shown in **Supplementary Figure 17D**.

**Figure 5:**
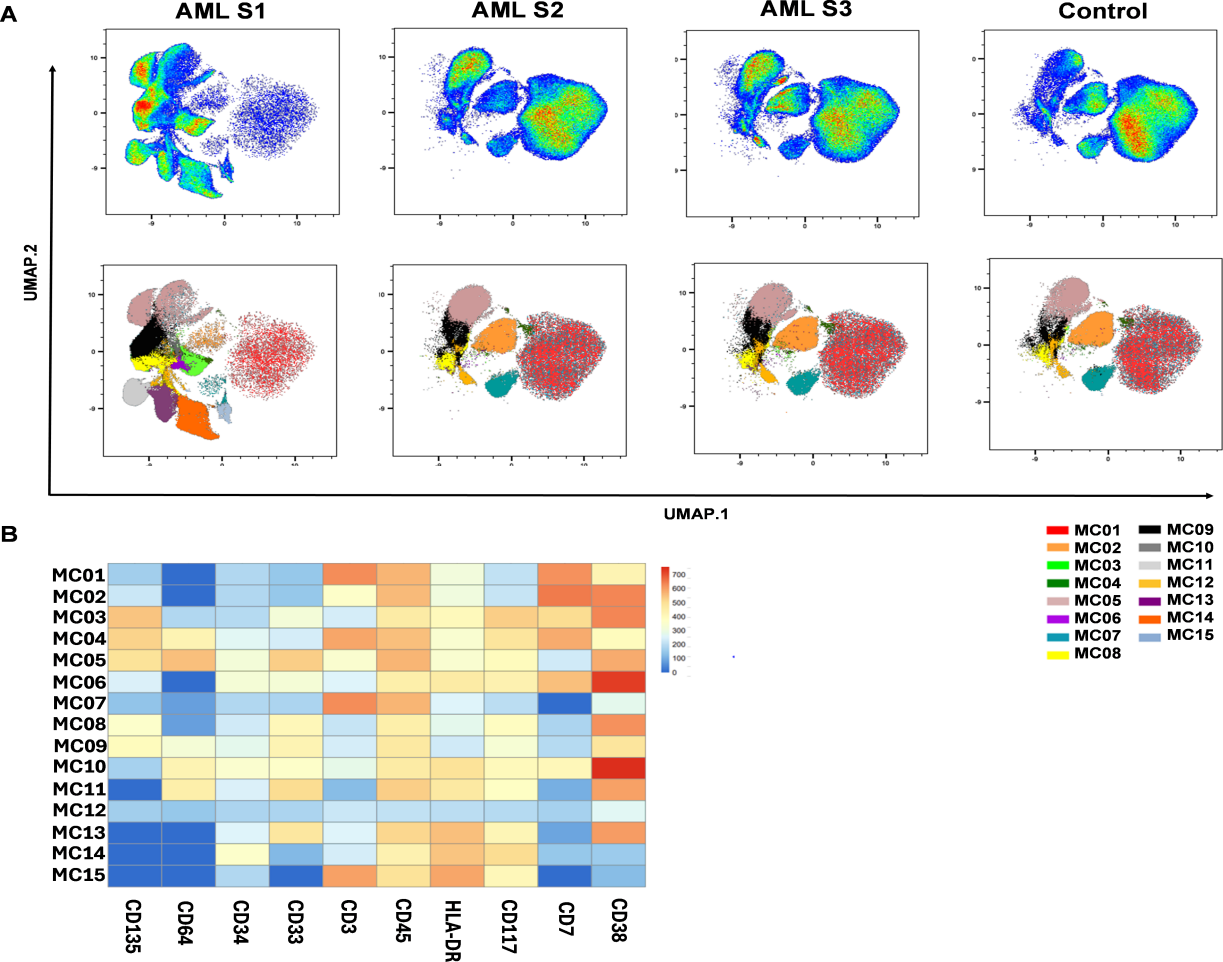
The normalization of leukemia-associated immunophenotype following therapy. **(A)** The UMAP visualization and FlowSOM analysis was performed including the follow-up samples to delineate the immune landscape of AML during the time-course of the follow-up interval. The upper row shows the UMAP cell density plot, the lower row shows the FlowSOM metacluster immune landscape on the UMAP cell clouds. Parental cell population for UMAP: CD19- living singlet (manually gated in FlowJo), cell number for UMAP: 22000/case; data were generated from 528,000 single cells from 24 cases (n=6 AML S1, n=6 AML S2, n=6 AML S3, n=6 HC). **(B)** The heatmap of metaclusters shows the marker expression intensities of each population. **(A, B)** Concatenated data of the 24 samples (n=6/group). The coloration is proportional with the extent of marker expression for blue (low expression) to red (high expression).

To follow the LAIP in an unsupervised manner and avoid human bias in manual gating, the UMAP visualization and FlowSOM analyses were carried out at time zero (S1) and two samplings following therapeutic regime application (S2 and S3) (**Figure 5**). The detailed therapeutic protocol and timing of peripheral blood withdrawal are summarized in **Supplementary Table 4**. Both the UMAP cell density plots and FlowSOM metacluster distribution plots showed the normalization of the LAIP following therapy (**Figure 5A**). The heatmap of the marker expression intensities of the follow-up samples and HC demonstrates the cell surface levels of the studied proteins in the MCs01-15 (**Figure 5B**).

**Figure 6:**
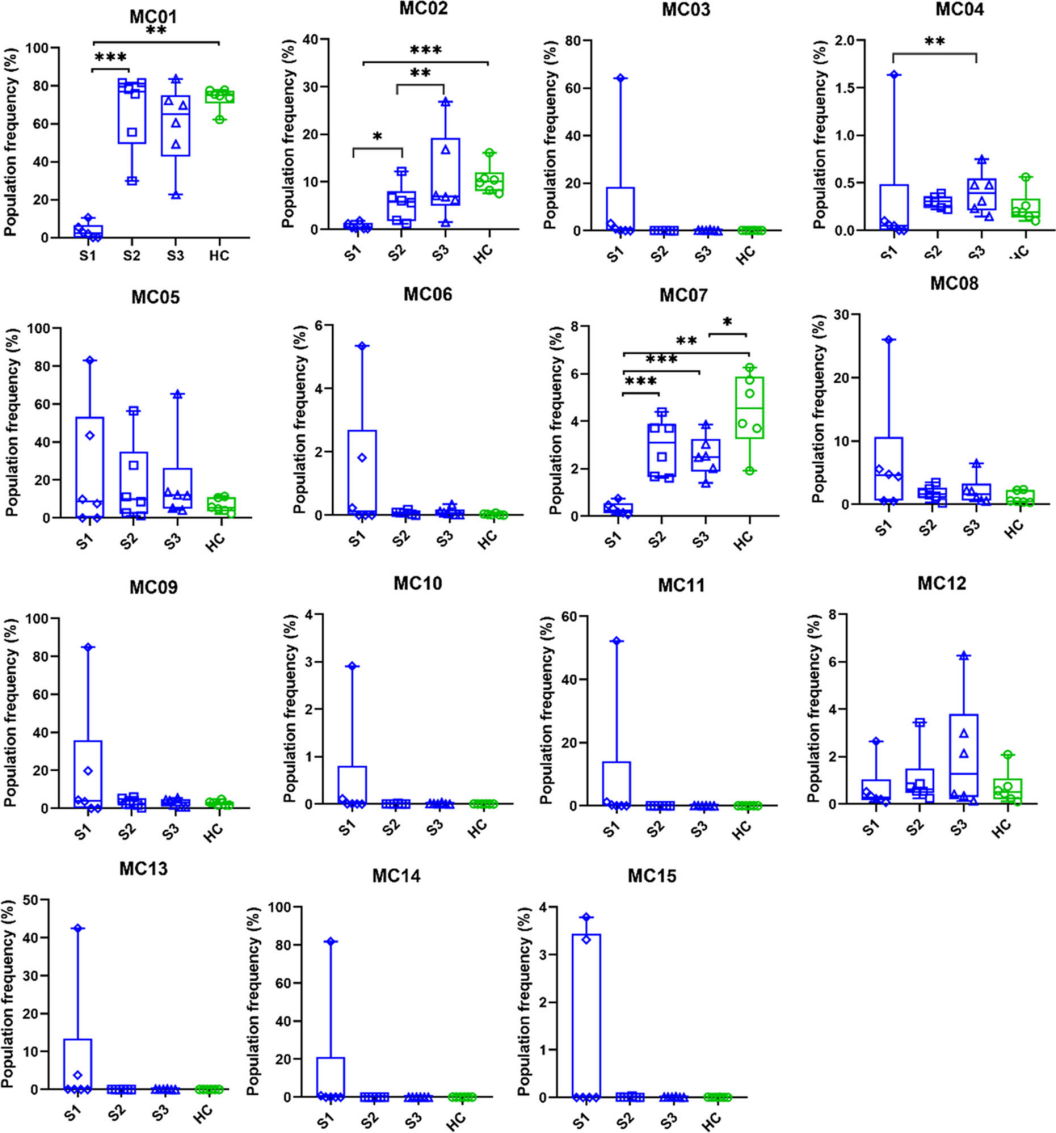
The population frequency changes of the metaclusters after treatments. The metacluster pattern of AML patients after treatment is increasingly similar to that of the healthy population. Differences are considered significant at *p < 0.05; **< 0.01; ***< 0.001. The option for Box and whiskers graphs were set in GraphPad Prism ‘min to max, show all points’. This method plots whiskers down to the minimum and up to the maximum value, but also plots each individual value as a point superimposed on the graph. The median values are also shown within the box by an equatorial line. The boxes are extended from the 25% percentile up to 75% percentile. Samples were paired with the corresponding age-and gender matched HC during the analysis. (n=6 AML S1, n=6 AML S2, n=6 AML S3, n=6 HC).

The follow-up of the individual MC distributions of the subjects is shown in **Supplementary Figure 18**. The patients still alive following the third sampling showed normalized MC frequency similar to that of the age- and sex-matched HC cases (**Supplementary Figure 18**). Next, to determine the most sensitive or resistant sub-populations to the treatment, the population frequencies of the 15 MCs were calculated (**Figure 6**). The emergence of MC01 (CD135-/CD64-/CD34-/CD33-/CD3+/CD45+/HLA-DRlow/CD117-/CD7+/CD38low), MC02 (CD135-/CD64-/CD34-/CD33-/CD3 low/CD45+/HLA-DRlow/CD117-/CD7+/CD38+), and MC07 (CD135-/CD64-/CD34-/CD33-/CD3+/CD45+/HLA-DR-/CD117-/CD7-/CD38-) significantly differentiated from S1 upon treatment and showed an increased level towards normalization, mainly the restoration of T-cell compartment. Other MCs decreased after treatment (MC03, MC04, MC05, MC06, MC08, MC09, MC10, MC11, MC13, MC14, MC15). Although these did not reach a significant difference, the tendency towards HC values suggested normalization. Only MC12 (CD135-/CD64-/CD34-/CD33-/CD3-/CD45-/HLA-DR-/CD117-/CD7-/CD38-) showed an increase upon treatment from 0.667% to 2.050% (arithmetic mean) by the 3^rd^ sampling during the follow-up (**Figure 6**).

### 3.3 Measurement of soluble mediators by the multiplex Luminex MAGPIX technology

After single-cell immunophenotyping of the cellular components of peripheral blood, our group focused on multiplex measurements of the soluble mediators, the concentrations of immuno-oncology checkpoint plasma proteins (32–34). Therefore, in addition to the cellular fraction of PBMCs, plasma samples were harvested. Six soluble mediators of the 31 in the panel showed significantly increased concentrations in AML vs. HCs: the BAFF (6482 vs. 569 pg/ml), B7-H2 (5849 vs. 2537 pg/ml), B7-H4 (437 vs. 292 pg/ml), CD25 (1547 vs. 656 pg/ml), MICA (30.9 vs. 20.1 pg/ml), and Siglec-7 (5.1 vs. 3.3 pg/ml). Three markers, CD40L (622 vs. 1068 pg/ml), E-cadherin (28061 vs. 54583 pg/ml), and soluble Gal-1 (4304 vs. 5221 pg/ml) decreased significantly in the plasma of peripheral blood of AML patients vs. HCs (**Supplementary Figure 19**).

During the follow-up of the patients, plasma concentrations of immuno-oncology mediators were also measured in parallel with FlowSOM-based monitoring of LAIP normalization. Five soluble mediators increased tendentiously upon treatment and reached values similar to those of HCs: E-cadherin, perforin, CD40L, B7-H6, and 5’NT/CD73 (**Supplementary Figure 20**). Three markers decreased tendentiously during treatment and reached normalized levels: BAFF, APRIL, and Siglec-9 (**Supplementary Figure 20**).

## 4 Discussion

The treatment of acute non-promyelocytic leukemia remains a serious challenge with poor outcomes despite the emergence of novel targeted agents (35). In the decision-making process of maintaining a balance between the toxicity of chemotherapy and the effectiveness of treatment, hematologists are in difficult situations during the management of AML. Flow cytometric assessment of AML phenotypes is of increasing clinical relevance in the era of immunotherapeutic strategies (36, 37). Knowledge of LAIP, different-from-normal (DfN) hematopoietic cell composition, and detection of MRD could assist in determining the appropriate intensity and necessity of the therapy, could help avoid overtreatment of patients, and lower the risk of unnecessary therapy-associated toxicity (38). The recent WHO2022 (5^th^ edition) and ICC classification of AML is mainly based on molecular, pathological, and cytogenetic data with high involvement of NGS, which requires expensive and time-consuming laboratory tests (39). Complementary to the recent diagnostic classification guidelines for detecting genetic alterations, MFC immunophenotyping may improve therapeutic decisions by providing fast, reliable, cost-effective, and locally understandable data. In the current study, a machine learning-assisted MFC was used to analyze the LAIP of AML cases before and after therapeutic intervention.

First, we demonstrated the applicability of our 12-plex FACS panel to differentiate the LAIP of therapy-naive AML from that of HCs by traditional manual gating. Five markers, CD33, CD34, CD38, CD117, and CD135, were significantly increased in the peripheral circulation of patients with AML. Next, the unsupervised UMAP was used to draw an AML-associated immune landscape versus HCs, and UMAP plots showed greater segmentation that reflects to greater population heterogeneity in AML vs HCs. In line with this, the FlowSOM algorithm identified eleven MCs with the characteristic of AML and only four MCs (MC01, MC06, MC07, and MC08) were significantly in higher rate in the HC group. Metaclusters that were present only in AML or that were in higher percentage in AML did not reach statistical significance. That can be explained with the interpatient heterogeneity of AML cases. Higher number of patients should be analyzed to reach statistical significance for AML specific metaclusters. The individual UMAP plots of the cases demonstrated a uniform immunophenotype of the investigated HCs in contrast to the unique immune landscape of the AML cases.

We showed restoration of the CD3 T-cell compartment after therapeutic intervention in the case of surviving patients. Additionally, CD7, CD33, CD34, CD38, CD64, CD117, CD135, and HLA-DR expression were monitored by manual gating during follow-up. A decrease in these markers, except for HLA-DR was observed in well-responder cases. We also proposed a gating strategy to define MRD cells as CD19 living singlets, CD45+, CD3-, CD38+/CD34-, CD38+/CD34+, CD117+/CD7+, and CD117+/CD135+, in line with previous MFC works (5, 6, 21, 40–43). The MFC has been reported to achieve up to 10^-4^ sensitivity in MRD detection (44), indeed, the initial AML blasts in the MRD gate were detectable at the lowest frequency in our current study of 22 cells per 100,000 (0.022%) CD45+CD3- living singlet parental population.

Using single-cell MFC and another round of FlowSOM and UMAP, we have shown normalization of the peripheral immunophenotype following therapy. We used UMAP and FlowSOM to identify therapy-sensitive and therapy-resistant subpopulations of patients with AML. Weijler et al. using two 8-membered antibody panels reported earlier the application of UMAP for the detection of MRD cells in AML (45). In the present study, unsupervised identification of therapy-resistant AML blasts in MC12 (CD3-CD7- CD33- CD38- CD64- HLA-DR- CD117- CD135-) using FlowSOM, or detection of MRD cells by manual gating, may represent important predictive markers of early relapse or long-term survival that could aid in more accurate selection of the appropriate therapy or populations needed for allo-HCT (46, 47). Recent publications highlighted the importance of leukemic stem cells with around 0.1-1% frequency, which play a crucial role in therapeutic resistance and recurrence (48–50). In the current study, the number of MC12 blasts increased by the 3^rd^ sampling during the follow-up period from min. 0.086-2.65% to max. 0.13-6.27% of CD19- living singlets. However, the authors speculate that MC12 cells negative for the investigated markers and expanding in the peripheral blood following treatment may represent leukemic stem cells in AML, but it would require additional investigations to validate.

The advantages of MFC, such as the fast, reliable, cost-effective and local delivery of the data within the clinical facility have made MFC widely available in hematology centers (51). However, the recent classification of AML based on mutational burden introduced genomics (NGS) at the forefront of AML diagnostics. The complementary nature of MFC and molecular pathology/genetics may reveal the most advanced diagnostic profile of AML patients (52). We propose that the combination of MFC immunophenotyping with the application of unsupervised evaluation methods such as UMAP and FlowSOM may support therapeutic decision-making in the future (53, 54).

To better understand the imbalance in immune homeostasis, Luminex MAGPIX technology was used to measure the concentration of 31 soluble immuno-oncology markers in the plasma of patients with AML. BAFF, B7-H2, B7-H4, CD25, MICA, and Siglec-7 levels were significantly increased in the plasma of AML subjects. The induction of BAFF in AML may be responsible for resistance to apoptosis (55), the production of B7-H2/ICOSL has been reported to promote the expansion of Tregs (56). B7-H4 has also been reported to have negative regulatory role in T-cell activation (57). The elevated serum concentrations of the soluble IL-2 receptor and CD25 have been described as a negative prognostic marker for chemotherapy in AML (58).

## 5 Conclusions

Taken together, (1) our group designed an antibody panel for studying AML, (2) our study sheds light on the utility of unsupervised multi-dimensional evaluation of single-cell immunophenotyping in therapy-naive AML. (3) The proposed MRD detection and, (4) the UMAP visualization and FlowSOM analysis of MFC data may serve for future diagnostics and prognosis of therapeutic response. (5) Multiparametric soluble marker detection may complement single-cell MFC immunophenotyping and enhance the characterization of immune dysregulation in AML. (6) In the future, the authors suggest integrating the AI-assisted MFC data evaluation, as a cloud based remote service for the clinical routine, that can facilitate the widespread availability of this technology.

## Data Availability

Raw data were generated at the Core Facility of HUN-REN Biological Research Centre, Laboratory of Functional Genomics. Derived data supporting the findings of this study are available from the corresponding author on request.

## References

[1] Abuelgasim KA Albuhayri B Munshi R Mugairi AA Alahmari B Gmati G. Impact of age and induction therapy on outcome of 180 adult patients with acute myeloid leukemia; retrospective analysis and literature review. Leuk Res Rep. (2020) 14:100206. doi: 10.1016/j.lrr.2020.100206 32566477 PMC7296329

[2] Almeida AM Ramos F. Acute myeloid leukemia in the older adults. Leuk Res Rep. (2016) 6:1–7. doi: 10.1016/j.lrr.2016.06.001 27408788 PMC4927655

[3] Huber S Baer C Hutter S Dicker F Meggendorfer M Pohlkamp C. AML classification in the year 2023: How to avoid a Babylonian confusion of languages. Leukemia. (2023) 37:1413–20. doi: 10.1038/s41375-023-01909-w PMC10317829 37120689

[4] Klepin HD. Definition of unfit for standard acute myeloid leukemia therapy. Curr Hematol Malig Rep. (2016) 11:537–44. doi: 10.1007/s11899-016-0348-8 27681539

[5] Heuser M Freeman SD Ossenkoppele GJ Buccisano F Hourigan CS Ngai LL. 2021 Update on MRD in acute myeloid leukemia: a consensus document from the European LeukemiaNet MRD Working Party. Blood. (2021) 138:2753–67. doi: 10.1182/blood.2021013626 PMC8718623 34724563

[6] Rohnert MA Kramer M SChadt J Ensel P Thiede C Krause SW. Reproducible measurable residual disease detection by multiparametric flow cytometry in acute myeloid leukemia. Leukemia. (2022) 36:2208–17. doi: 10.1038/s41375-022-01647-5 PMC9417981 35851154

[7] Othman J Potter N Ivey A Jovanovic J Runglall M Freeman SD. Postinduction molecular MRD identifies patients with NPM1 AML who benefit from allogeneic transplant in first remission. Blood. (2024) 143:1931–6. doi: 10.1182/blood.2023023096 38364112

[8] Loke J Buka R Craddock C. Allogeneic stem cell transplantation for acute myeloid leukemia: who, when, and how? Front Immunol. (2021) 12:659595. doi: 10.3389/fimmu.2021.659595 34012445 PMC8126705

[9] Voso MT Ottone T Lavorgna S Venditti A Maurillo L Lo-Coco F. MRD in AML: the role of new techniques. Front Oncol. (2019) 9:655. doi: 10.3389/fonc.2019.00655 31396481 PMC6664148

[10] Schuurhuis GJ Heuser M Freeman S Bene MC Buccisano F Cloos J. Minimal/measurable residual disease in AML: a consensus document from the European LeukemiaNet MRD Working Party. Blood. (2018) 131:1275–91. doi: 10.1182/blood-2017-09-801498. PubMed PMID: 29330221; PubMed Central PMCID: PMCPMC5865231 project) from Beckman Coulter. J.C. received research funding from Helsinn Healthcare, Janssen Pharmaceuticals, Merus, and Takeda. S.F. received support from National Institute for Health Research, CRUK, and Bloodwise. T.H. and W.K. @ are both part owners of Munich Leukemia Laboratory. C.S.H. received research funding from Merck and Sellas. G.J.O. provided consultancy services to Janssen and Sunesis; served on the advisory board for Novartis, Pfizer, BMS, Janssen, Sunesis, Celgene, Karyopharm, Amgen, and Seattle Genetics; and received research funding from Novartis, Janssen, Celgene, Immunogen, and Becton Dickinson. G.J.R. provided consultancy services to AbbVie, Amgen, Amphivena Therapeutics, Astex Pharmaceuticals, Array BioPharma Inc., Celgene, Clovis Oncology, CTI BioPharma, Genoptix, Immune Pharmaceuticals, Janssen Pharmaceutica, Jazz Pharmaceuticals, Juno Therapeutics, MedImmune, Novartis, Onconova Therapeutics, Orsenix, Pfizer, Roche/Genentech, and Sunesis Pharmaceuticals and received research support from Cellectis. G.J.S. received research funding from Novartis, Janssen, Immunogen, and Becton Dickinson. C.T. @ is part Chief Research Officer and Chief Executive Officer and owner of AgenDix GmbH, a company performing molecular diagnostics. The remaining authors declare no competing financial interests. PMC5865231 29330221

[11] Del Nagro CJ Otero DC Anzelon AN Omori SA Kolla RV Rickert RC. CD19 function in central and peripheral B-cell development. Immunol Res. (2005) 31:119–31. doi: 10.1385/IR:31:2:119 15778510

[12] Rojo JM Bello R Portoles P. T-cell receptor. Adv Exp Med Biol. (2008) 640:1–11. doi: 10.1007/978-0-387-09789-3_1 19065779

[13] Ehninger A Kramer M Rollig C Thiede C Bornhauser M von Bonin M. Distribution and levels of cell surface expression of CD33 and CD123 in acute myeloid leukemia. Blood Cancer J. (2014) 4:e218. doi: 10.1038/bcj.2014.39 24927407 PMC4080210

[14] Tsuji K Feng MA Wang D. Development of human lymphohematopoiesis defined by CD34 and CD81 expression. Leuk Lymphoma. (2002) 43:2269–73. doi: 10.1080/1042819021000039974 12613512

[15] Gorczyca W Sun ZY Cronin W Li X Mau S Tugulea S. Immunophenotypic pattern of myeloid populations by flow cytometry analysis. Methods Cell Biol. (2011) 103:221–66. doi: 10.1016/B978-0-12-385493-3.00010-3 21722806

[16] Gajendra S Gupta R Thakral D Gupta SK Jain G Bakhshi S. CD34 negative HLA-DR negative acute myeloid leukemia: A higher association with NPM1 and FLT3-ITD mutations. Int J Lab Hematol. (2023) 45:221–8. doi: 10.1111/ijlh.14007 36504282

[17] Li W Liang L Liao Q Li Y Zhou Y. CD38: An important regulator of T cell function. BioMed Pharmacother. (2022) 153:113395. doi: 10.1016/j.biopha.2022.113395 35834988

[18] Dunphy CH Tang W. The value of CD64 expression in distinguishing acute myeloid leukemia with monocytic differentiation from other subtypes of acute myeloid leukemia: a flow cytometric analysis of 64 cases. Arch Pathol Lab Med. (2007) 131:748–54. doi: 10.5858/2007-131-748-TVOCEI 17488160

[19] El-Meligui YM Abd Elrhman HE Salahuddin A Hamouda MA Kassem AB. Correlation study on HLA-DR and CD117 (c-kit) expressions: its prognosis and treatment response in acute myeloid leukemia patients. Pharmgenomics Pers Med. (2021) 14:381–93. doi: 10.2147/PGPM.S268986 PMC8019664 33833549

[20] Kazi JU Ronnstrand L. FMS-like tyrosine kinase 3/FLT3: from basic science to clinical implications. Physiol Rev. (2019) 99:1433–66. doi: 10.1152/physrev.00029.2018 31066629

[21] Kandeel EZ El Sayed G Elsharkawy N Eldin DN Nassar HR Ibrahiem D. Impact of FLT3 receptor (CD135) detection by flow cytometry on clinical outcome of adult acute myeloid leukemia patients. Clin Lymphoma Myeloma Leuk. (2018) 18:541–7. doi: 10.1016/j.clml.2018.05.014 29907544

[22] Roerden M Marklin M Salih HR Bethge WA Klein R Rammensee HG. Expression levels of HLA-DR in acute myeloid leukemia: implications for antigenicity and clinical outcome. Leuk Lymphoma. (2021) 62:1907–19. doi: 10.1080/10428194.2021.1885659 33648413

[23] Khoury JD Solary E Abla O Akkari Y Alaggio R Apperley JF. The 5th edition of the world health organization classification of hematolymphoid tumors: myeloid and histiocytic/dendritic neoplasms. Leukemia. (2022) 36:1703–19. doi: 10.1038/s41375-022-01613-1 PMC9252913 35732831

[24] Narayanan D Weinberg OK. How I investigate acute myeloid leukemia. Int J Lab Hematol. (2020) 42:3–15. doi: 10.1111/ijlh.13135 31820579

[25] Walter RB Othus M Burnett AK Lowenberg B Kantarjian HM Ossenkoppele GJ. Significance of FAB subclassification of "acute myeloid leukemia, NOS" in the 2008 WHO classification: analysis of 5848 newly diagnosed patients. Blood. (2013) 121:2424–31. doi: 10.1182/blood-2012-10-462440 PMC3612855 23325837

[26] Krasinskas AM Wasik MA Kamoun M Schretzenmair R Moore J Salhany KE. The usefulness of CD64, other monocyte-associated antigens, and CD45 gating in the subclassification of acute myeloid leukemias with monocytic differentiation. Am J Clin Pathol. (1998) 110:797–805. doi: 10.1093/ajcp/110.6.797 9844593

[27] Tisone JA Bohman JE Theil KS Brandt JT. Aberrant expression of CD19 as a marker of monocytic lineage in acute myelogenous leukemia. Am J Clin Pathol. (1997) 107:283–91. doi: 10.1093/ajcp/107.3.283 9052378

[28] Szabo E Modok S Ronaszeki B Farago A Gemes N Nagy LI. Comparison of humoral and cellular immune responses in hematologic diseases following completed vaccination protocol with BBIBP-CorV, or AZD1222, or BNT162b2 vaccines against SARS-CoV-2. Front Med (Lausanne). (2023) 10:1176168. doi: 10.3389/fmed.2023.1176168 37529238 PMC10389666

[29] Honfi D Gemes N Szabo E Neuperger P Balog JA Nagy LI. Comparison of homologous and heterologous booster SARS-coV-2 vaccination in autoimmune rheumatic and musculoskeletal patients. Int J Mol Sci. (2022) 23(19):11411. doi: 10.3390/ijms231911411 36232710 PMC9569441

[30] Becht E McInnes L Healy J Dutertre CA Kwok IWH Ng LG. Dimensionality reduction for visualizing single-cell data using UMAP. Nat Biotechnol. (2018) 37:38–44. doi: 10.1038/nbt.4314 30531897

[31] Van Gassen S Callebaut B Van Helden MJ Lambrecht BN Demeester P Dhaene T. FlowSOM: Using self-organizing maps for visualization and interpretation of cytometry data. Cytometry A. (2015) 87:636–45. doi: 10.1002/cyto.a.22625 25573116

[32] Balog JA Kemeny A Puskas LG Burcsar S Balog A Szebeni GJ. Investigation of newly diagnosed drug-naive patients with systemic autoimmune diseases revealed the cleaved peptide tyrosine tyrosine (PYY 3-36) as a specific plasma biomarker of rheumatoid arthritis. Mediators Inflammation. (2021) 2021:5523582. doi: 10.1155/2021/5523582 PMC8240466 34239365

[33] Toth ME Dukay B Peter M Balogh G Szucs G Zvara A. Male and female animals respond differently to high-fat diet and regular exercise training in a mouse model of hyperlipidemia. Int J Mol Sci. (2021) 22(8):4198. doi: 10.3390/ijms22084198 PMC8073713 33919597

[34] Gemes N Balog JA Neuperger P Schlegl E Barta I Fillinger J. Single-cell immunophenotyping revealed the association of CD4+ central and CD4+ effector memory T cells linking exacerbating chronic obstructive pulmonary disease and NSCLC. Front Immunol. (2023) 14:1297577. doi: 10.3389/fimmu.2023.1297577 38187374 PMC10770259

[35] Abou Dalle I Atoui A Bazarbachi A. The elephant in the room: AML relapse post allogeneic hematopoietic cell transplantation. Front Oncol. (2021) 11:793274. doi: 10.3389/fonc.2021.793274 35047405 PMC8761806

[36] Ally F Chen X. Acute myeloid leukemia: diagnosis and evaluation by flow cytometry. Cancers (Basel). (2024) 16(22):3855. doi: 10.3390/cancers16223855 39594810 PMC11592599

[37] Wu Y Li Y Gao Y Zhang P Jing Q Zhang Y. Immunotherapies of acute myeloid leukemia: Rationale, clinical evidence and perspective. BioMed Pharmacother. (2024) 171:116132. doi: 10.1016/j.biopha.2024.116132 38198961

[38] Gutman JA Winters A Kent A Amaya M McMahon C Smith C. Higher-dose venetoclax with measurable residual disease-guided azacitidine discontinuation in newly diagnosed acute myeloid leukemia. Haematologica. (2023) 108:2616–25. doi: 10.3324/haematol.2023.282681 PMC10542846 37051756

[39] Park HS. What is new in acute myeloid leukemia classification? Blood Res. (2024) 59:15. doi: 10.1007/s44313-024-00016-8 38616211 PMC11016528

[40] Wood BL. Principles of minimal residual disease detection for hematopoietic neoplasms by flow cytometry. Cytometry B Clin Cytom. (2016) 90:47–53. doi: 10.1002/cyto.b.21239 25906832

[41] Weeda V Mestrum SGC Leers MPG. Flow cytometric identification of hematopoietic and leukemic blast cells for tailored clinical follow-up of acute myeloid leukemia. Int J Mol Sci. (2022) 23(18):10529. doi: 10.3390/ijms231810529 36142442 PMC9506284

[42] Moritz J Schwab A Reinisch A Zebisch A Sill H Wolfler A. Measurable residual disease detection in acute myeloid leukemia: current challenges and future directions. Biomedicines. (2024) 12(3):599. doi: 10.3390/biomedicines12030599 38540210 PMC10968436

[43] Chen M Fu M Gong M Gao Y Wang A Zhao W. Twenty-four-color full spectrum flow cytometry panel for minimal residual disease detection in acute myeloid leukemia. Open Med (Wars). (2023) 18:20230745. doi: 10.1515/med-2023-0745 37533738 PMC10390751

[44] Kantarjian H Borthakur G Daver N DiNardo CD Issa G Jabbour E. Current status and research directions in acute myeloid leukemia. Blood Cancer J. (2024) 14:163. doi: 10.1038/s41408-024-01143-2. PubMed PMID: 39300079; PubMed Central PMCID: PMCPMC11413327 Daiichi-Sankyo, Immunogen, Novartis; honoraria from Ipsen Biopharmaceuticals, KAHR Medical, Shenzhen Target Rx, Stemline, Takeda. TK reports grant or research support from BMS, Celgene, Pfizer, Amgen, Jazz, AstraZeneca and Genentech; consultant fees from Agios, Jazz, Genentech and Novartis. CDiN reports research support to institution from Abbvie, Agios, Bayer, Calithera, Cleave, BMS/Celgene, Daiichi-Sankyo and ImmuneOnc; consultant/advisory boards with Abbvie, Agios, Celgene/BMS, Daiichi-Sankyo, ImmuneOnc, Novartis, Takeda and Notable Labs. ND reports research funding from Daiichi-Sankyo, Bristol-Myers Squibb, Pfizer, Gilead, Sevier, Genentech, Astellas, Daiichi-Sankyo, Abbvie, Hanmi, Trovagene, FATE, Amgen, Novimmune, Glycomimetics, and ImmunoGen and has served in a consulting or advisory role for Daiichi-Sankyo, Bristol-Myers Squibb, Pfizer, Novartis, Celgene, AbbVie, Astellas, Genentech, Immunogen, Servier, Syndax, Trillium, Gilead, Amgen and Agios. GB reports research funding from Bristol-Myers Squibb, GlaxoSmithKline, Janssen Scientific Affairs, Eli Lilly and Company, Cyclacel, AstraZeneca, AbbVie, Oncoceutics, Arvinas, Cantargia, PTC Therapeutics, Argenx, BioTheryX, and Bioline and personal fees from PTC Therapeutics, Argenx, BioTheryX, and Bioline. GI reports research funding from Celgene, Merck, Kura Oncology, Syndax, Astex and Novartis, and received consultancy or advisory board fees from NuProbe, AbbVie, Novartis, Sanofi, AstraZeneca, Syndax and Kura Oncology. EJ reports research grants and advisory rolls with AbbVie, Adaptive Biotechnologies, Amgen, BMS, Pfizer and Takeda, and advisory role with Genetech. FR reports research funding from BMS, Amgen, Xencor, Macrogenics, Orsenix, Abbvie, Prelude, Astex; consultancy and honoraria from Celgene, BMS, Amgen, Astellas, Xencor, Agios, AstraZeneca and Orsenix. 39300079 PMC11413327

[45] Weijler L Kowarsch F Wodlinger M Reiter M Maurer-Granofszky M Schumich A. UMAP based anomaly detection for minimal residual disease quantification within acute myeloid leukemia. Cancers (Basel). (2022) 14(4):898. doi: 10.3390/cancers14040898 35205645 PMC8870142

[46] Dekker SE Rea D Cayuela JM Arnhardt I Leonard J Heuser M. Using measurable residual disease to optimize management of AML, ALL, and chronic myeloid leukemia. Am Soc Clin Oncol Educ Book. (2023) 43:e390010. doi: 10.1200/EDBK_390010 37311155

[47] Shimony S Stahl M Stone RM. Acute myeloid leukemia: 2023 update on diagnosis, risk-stratification, and management. Am J Hematol. (2023) 98:502–26. doi: 10.1002/ajh.26822 36594187

[48] Long NA Golla U Sharma A Claxton DF. Acute myeloid leukemia stem cells: origin, characteristics, and clinical implications. Stem Cell Rev Rep. (2022) 18:1211–26. doi: 10.1007/s12015-021-10308-6 PMC10942736 35050458

[49] Chen Y Li J Xu L Gaman MA Zou Z. The genesis and evolution of acute myeloid leukemia stem cells in the microenvironment: From biology to therapeutic targeting. Cell Death Discovery. (2022) 8:397. doi: 10.1038/s41420-022-01193-0 36163119 PMC9513079

[50] Stelmach P Trumpp A. Leukemic stem cells and therapy resistance in acute myeloid leukemia. Haematologica. (2023) 108:353–66. doi: 10.3324/haematol.2022.280800 PMC9890038 36722405

[51] Chen X Cherian S. Acute myeloid leukemia immunophenotyping by flow cytometric analysis. Clin Lab Med. (2017) 37:753–69. doi: 10.1016/j.cll.2017.07.003 29128067

[52] Pessoa F MaChado CB Barreto IV Sampaio GF Oliveira DS Ribeiro RM. Association between immunophenotypic parameters and molecular alterations in acute myeloid leukemia. Biomedicines. (2023) 11(4):1098. doi: 10.3390/biomedicines11041098 37189716 PMC10135936

[53] Cheng FM Lo SC Lin CC Lo WJ Chien SY Sun TH. Deep learning assists in acute leukemia detection and cell classification via flow cytometry using the acute leukemia orientation tube. Sci Rep. (2024) 14:8350. doi: 10.1038/s41598-024-58580-z 38594383 PMC11004172

[54] Lewis JE Cooper LAD Jaye DL Pozdnyakova O. Automated deep learning-based diagnosis and molecular characterization of acute myeloid leukemia using flow cytometry. Mod Pathol. (2024) 37:100373. doi: 10.1016/j.modpat.2023.100373 37925056

[55] Bolkun L Grubczak K Schneider G Zembko P Radzikowska U Singh P. Involvement of BAFF and APRIL in resistance to apoptosis of acute myeloid leukemia. J Cancer. (2016) 7:1979–83. doi: 10.7150/jca.15966 PMC5118661 27877213

[56] Han Y Dong Y Yang Q Xu W Jiang S Yu Z. Acute myeloid leukemia cells express ICOS ligand to promote the expansion of regulatory T cells. Front Immunol. (2018) 9:2227. doi: 10.3389/fimmu.2018.02227 30319662 PMC6168677

[57] Wang JY Wang WP. B7-H4, a promising target for immunotherapy. Cell Immunol. (2020) 347:104008. doi: 10.1016/j.cellimm.2019.104008 31733822

[58] Nakase K Kita K Kyo T Tsuji K Katayama N. High serum levels of soluble interleukin-2 receptor in acute myeloid leukemia: correlation with poor prognosis and CD4 expression on blast cells. Cancer Epidemiol. (2012) 36:e306–9. doi: 10.1016/j.canep.2012.03.011 22537764

