# Supplementary material for: Multiplex immunophenotyping of human acute myeloid leukemia patients revealed single -cell heterogeneity with special attention on therapy sensitive and therapy resistant subpopulations
Source: Front Immunol. 2025 Apr 17;16:1563386. doi: 10.3389/fimmu.2025.1563386 (PMC12043712; doi:10.3389/fimmu.2025.1563386)
Supplement: Supplementary file 1 [file DataSheet1.pdf]

Figure S1.

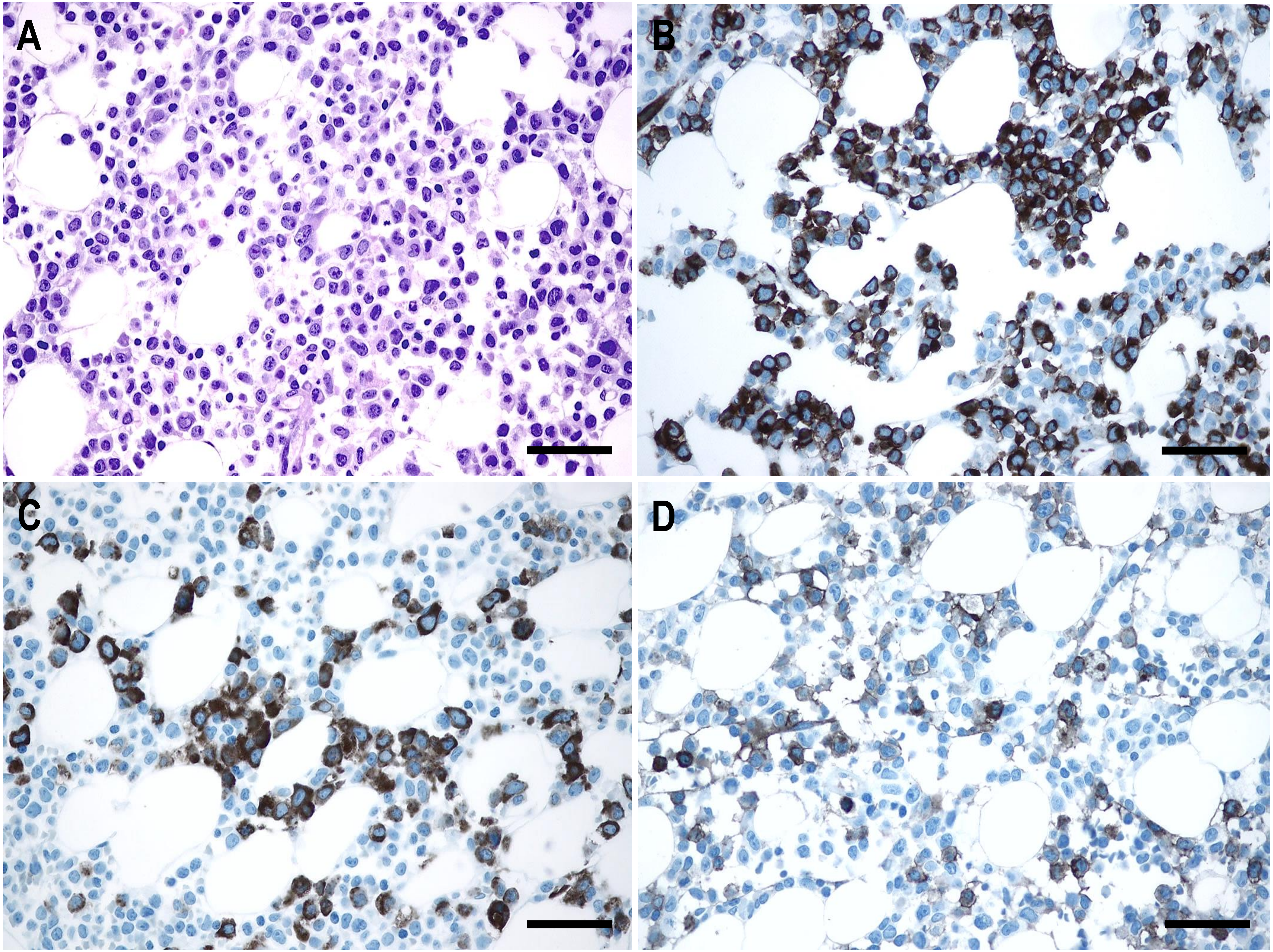


IHC of AML4

Figure S2.

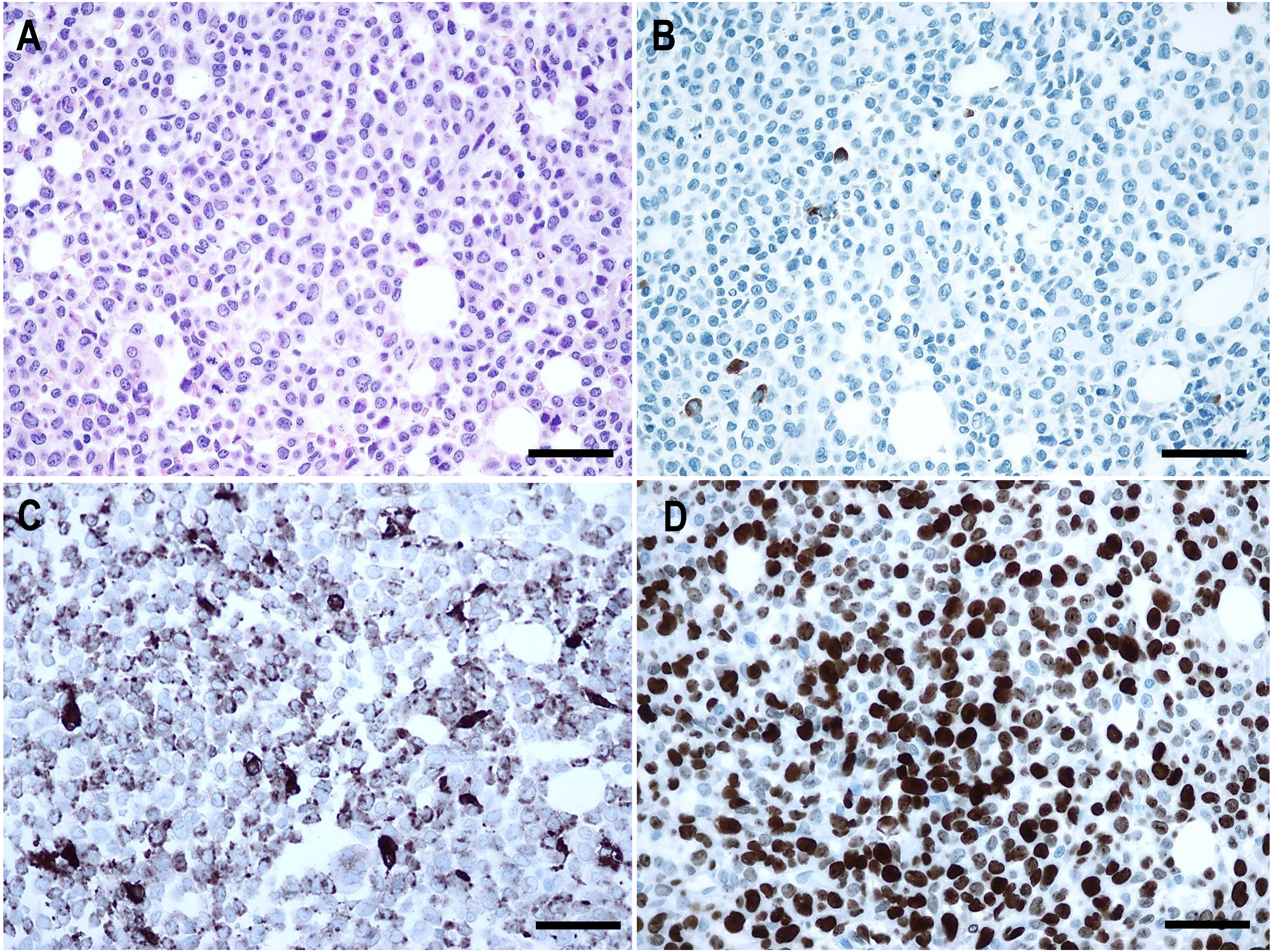


IHC of AML5.

Figure S3.

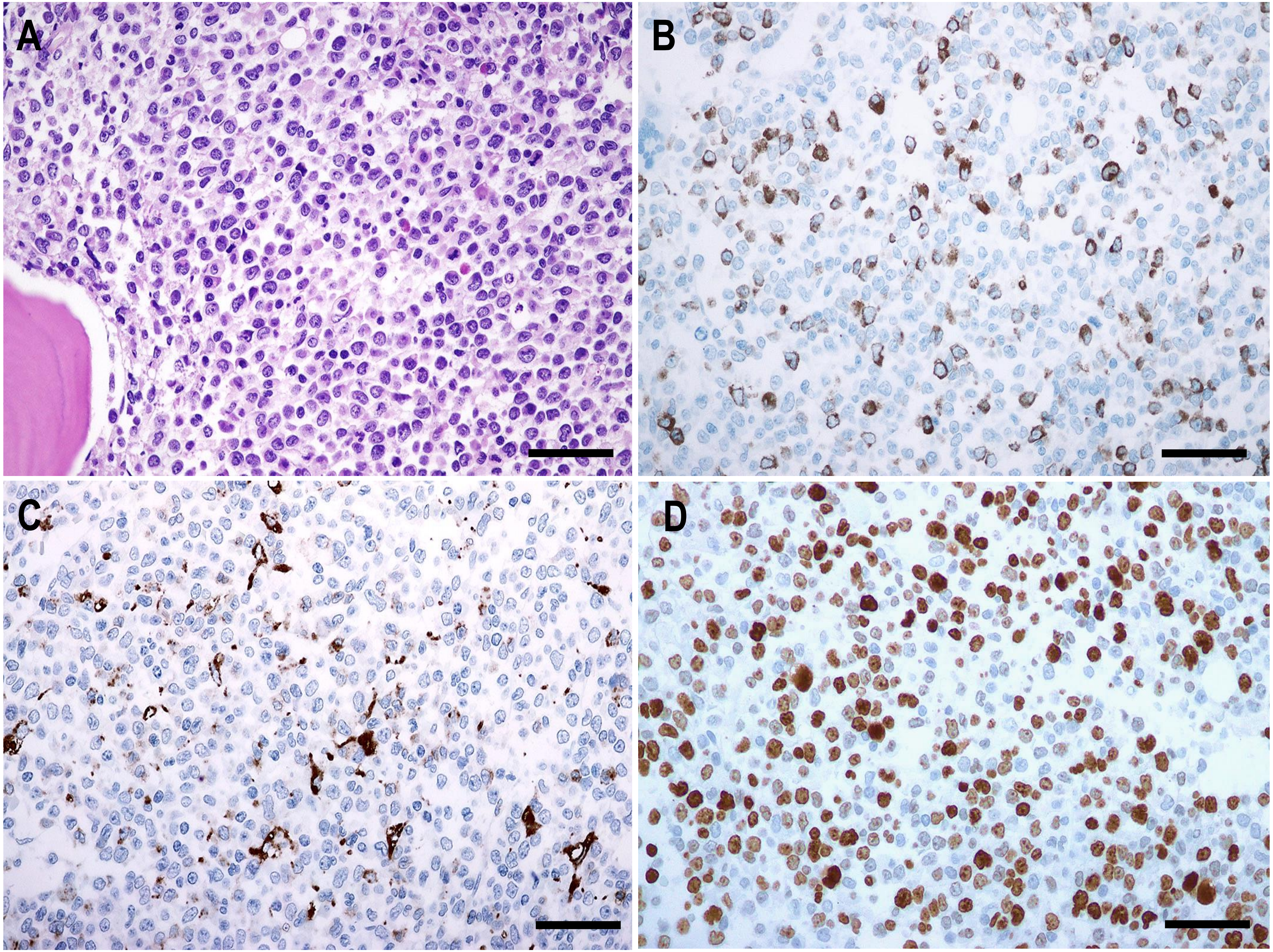


IHC of AML6.

Figure S4.

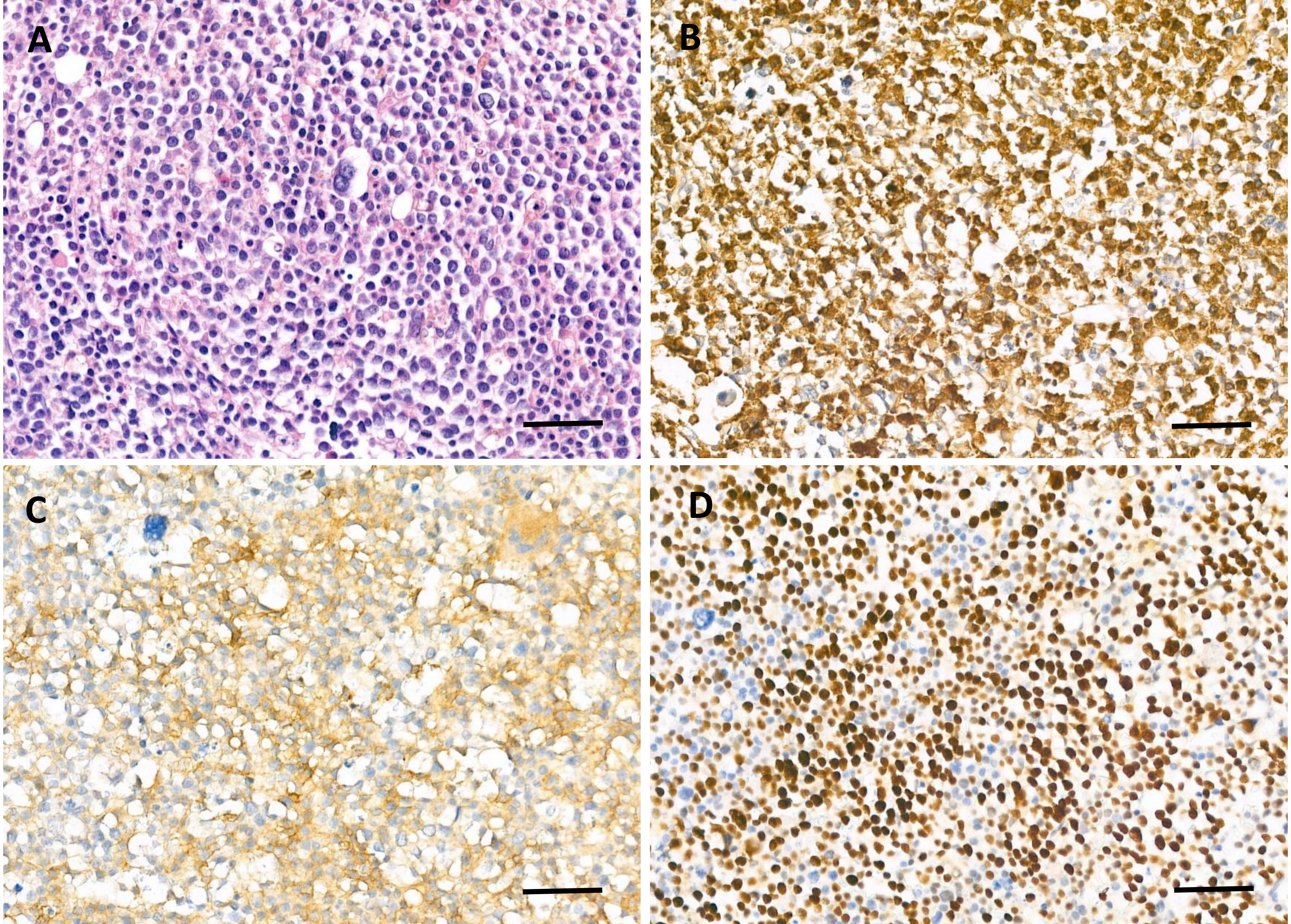


IHC of AML7.

Figure S5.

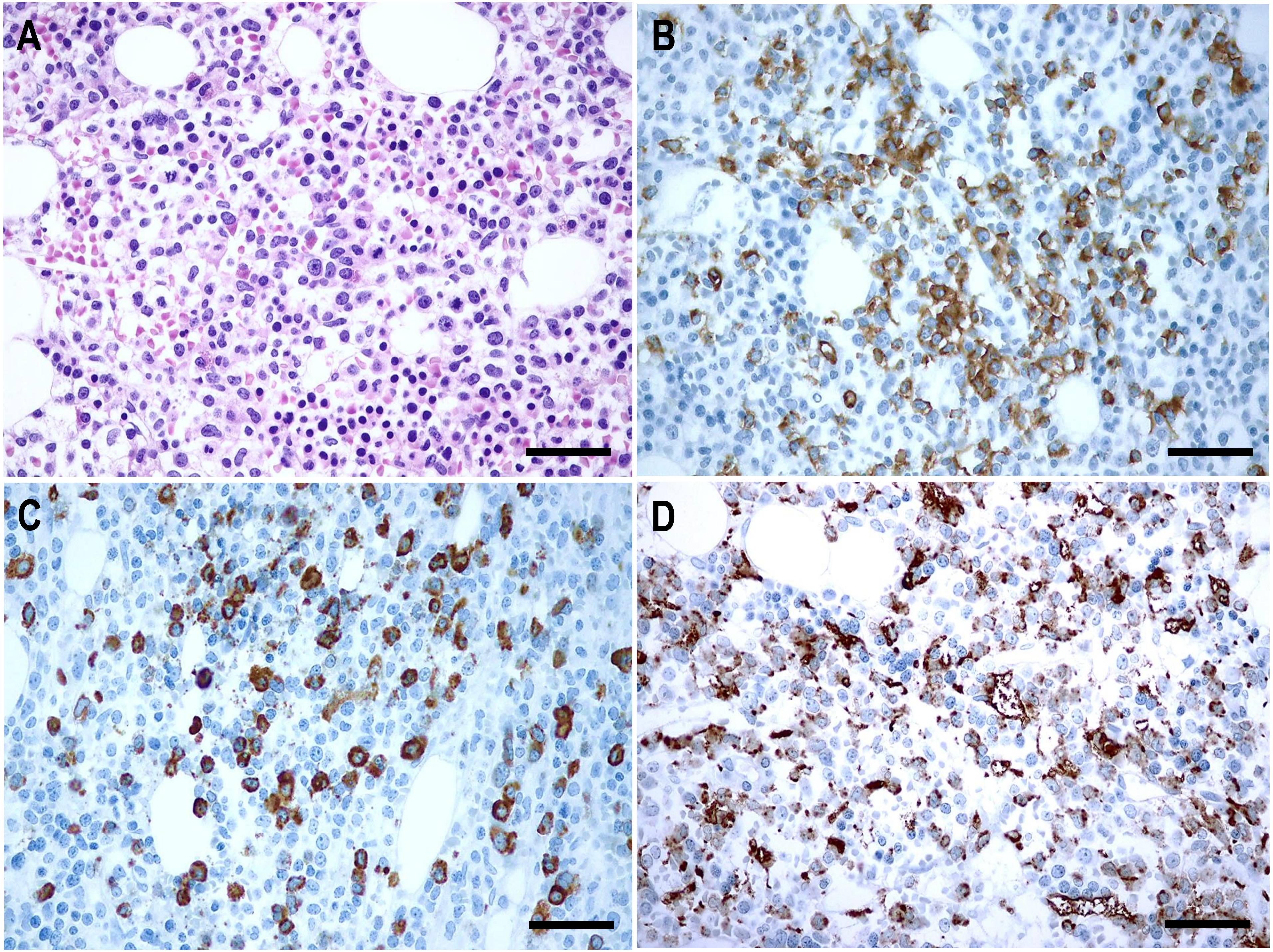


IHC of AML8.

Figure S6.

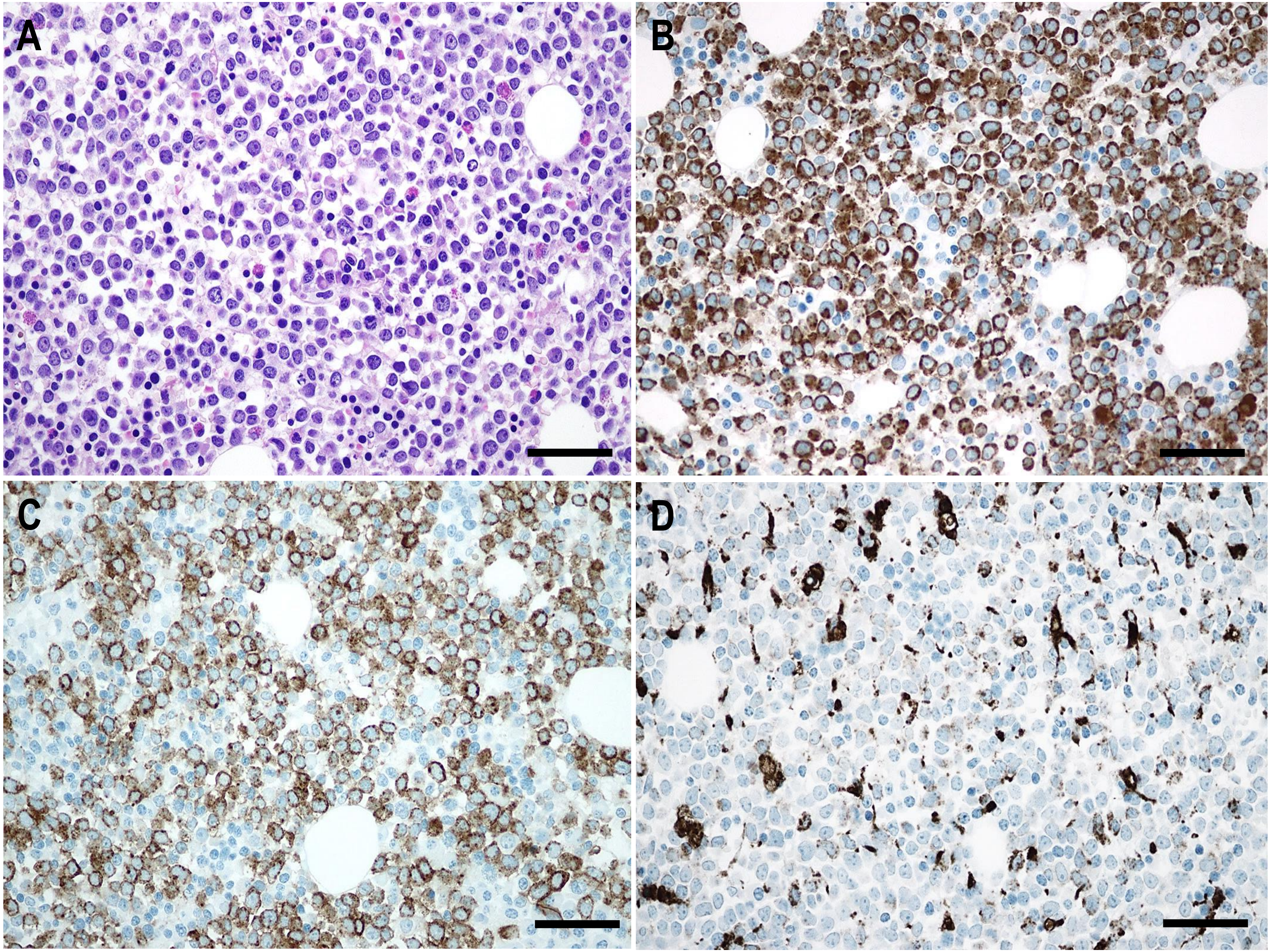


IHC of AML9.

Figure S7.

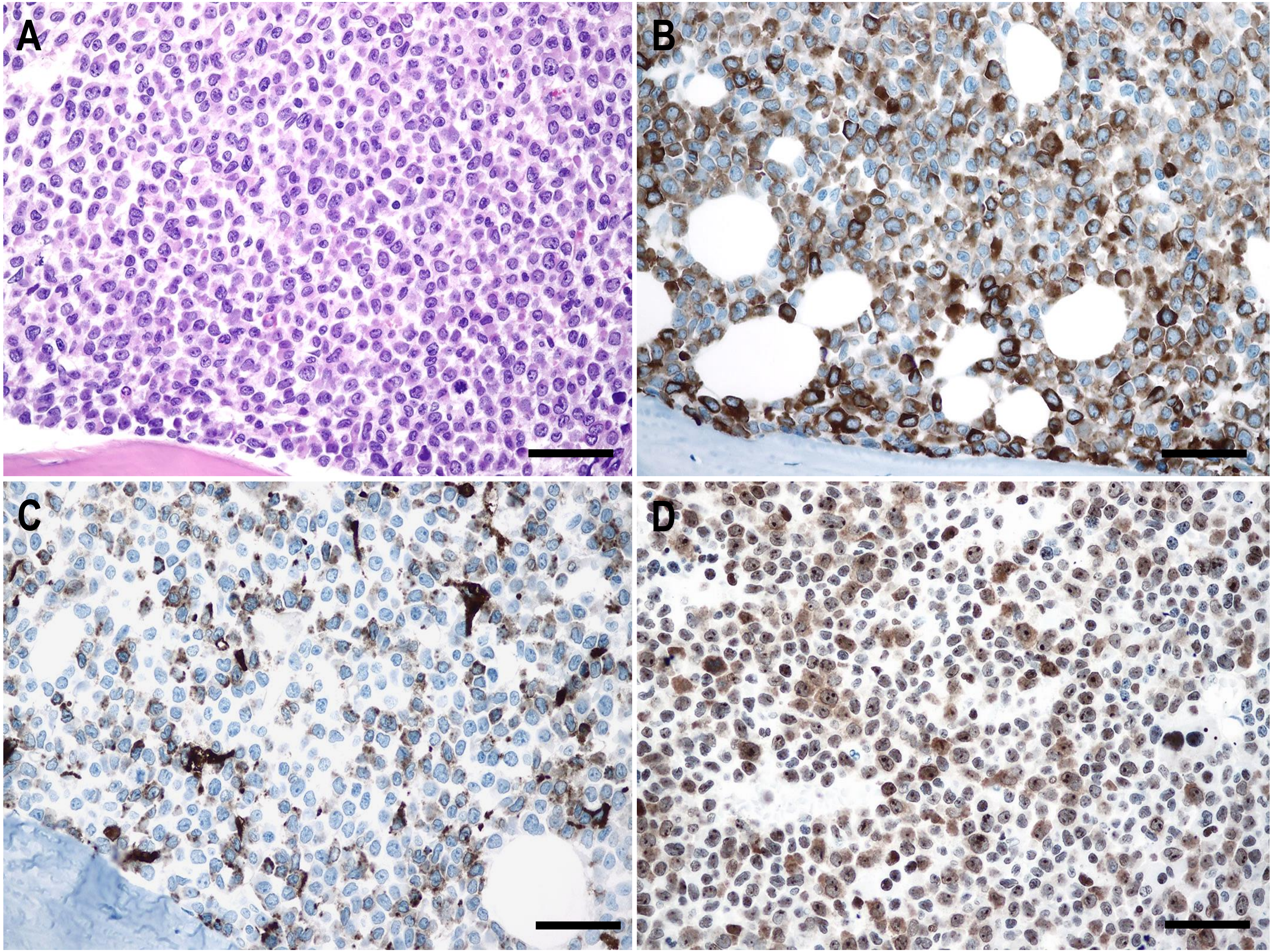


IHC of AML10.

**The IHC of AML11 was not possible to perform.**

Figure S8.

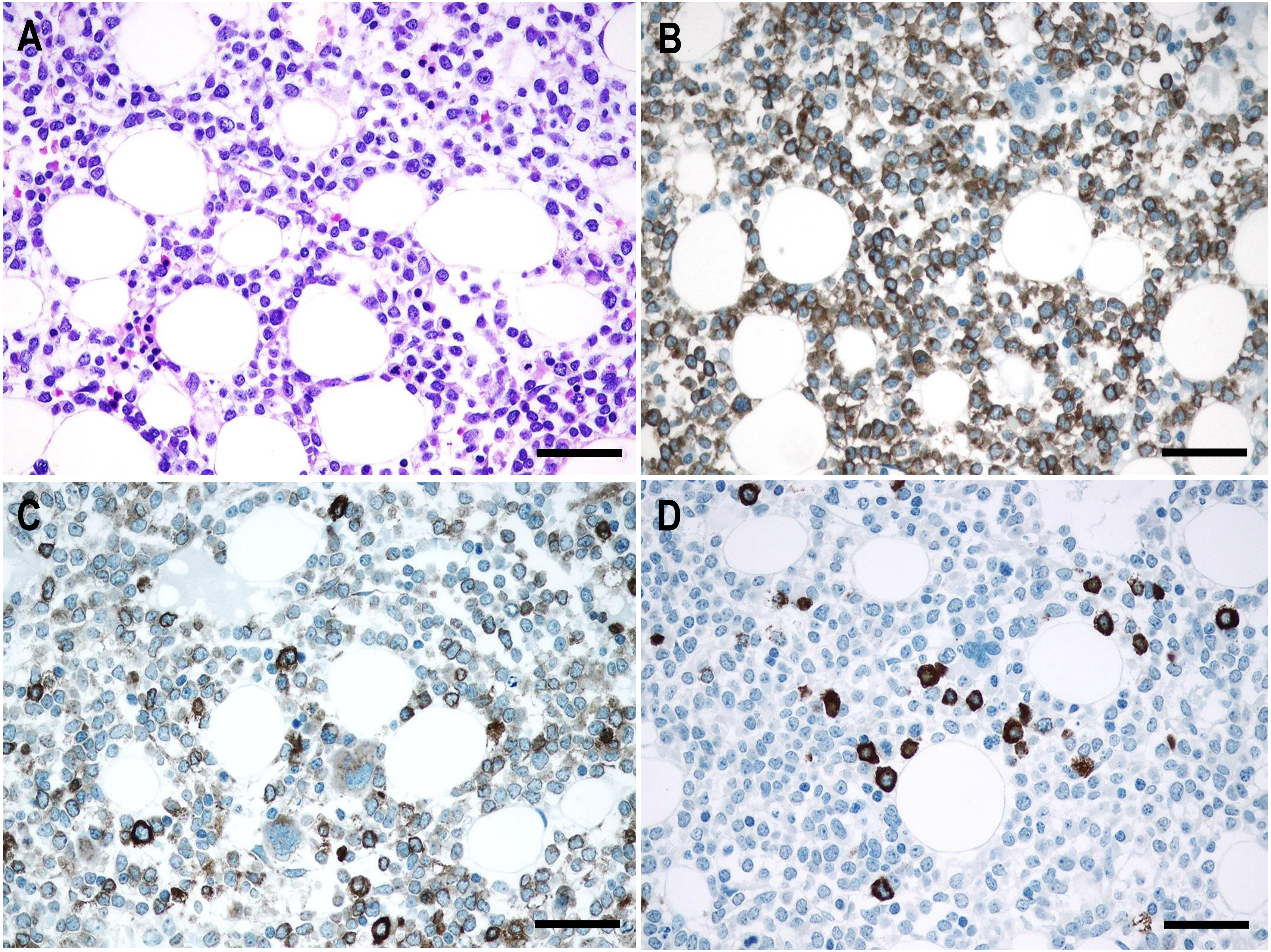


IHC of AML12.

Figure S9.

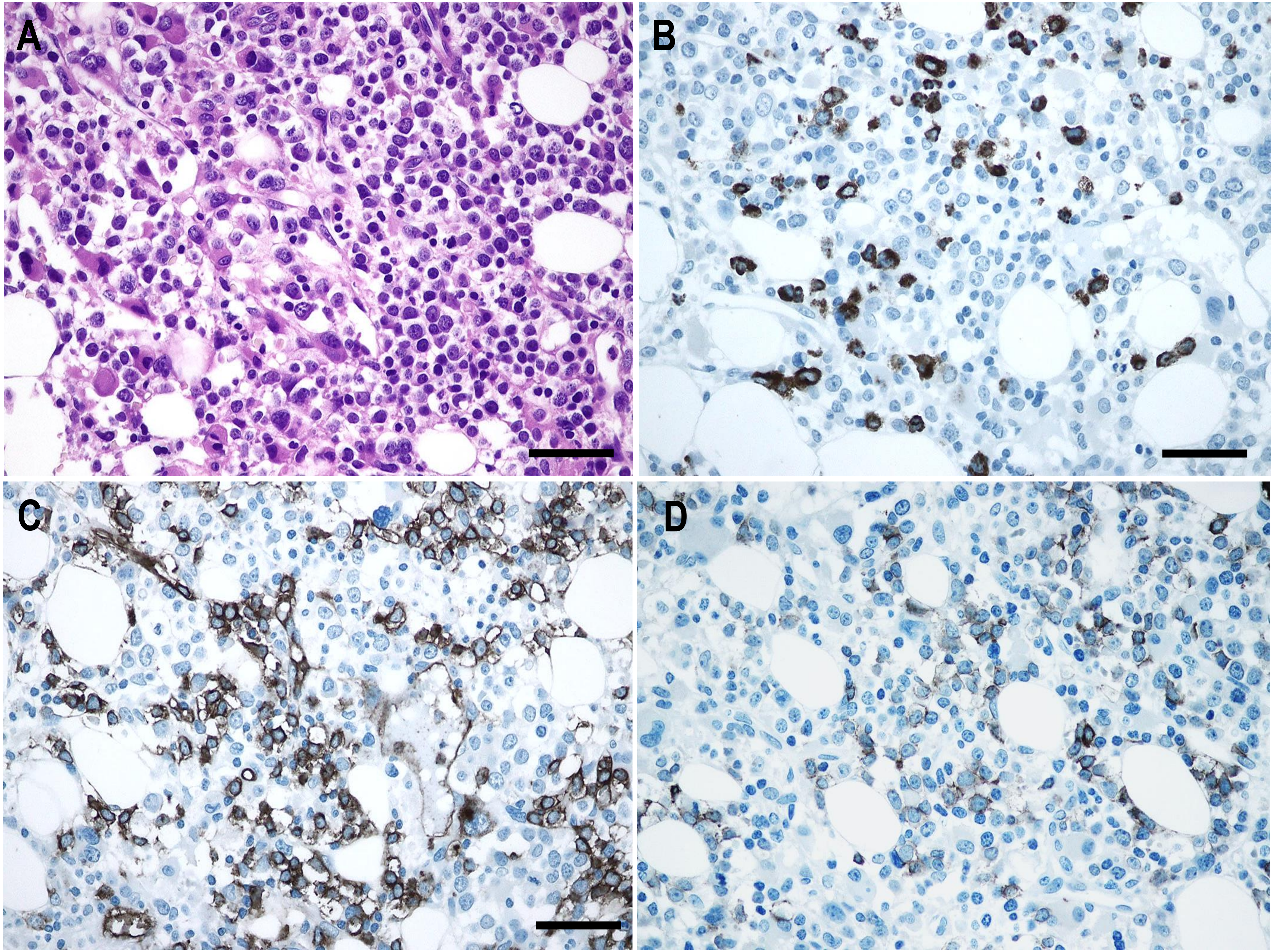


IHC of AML13.

Figure S10.

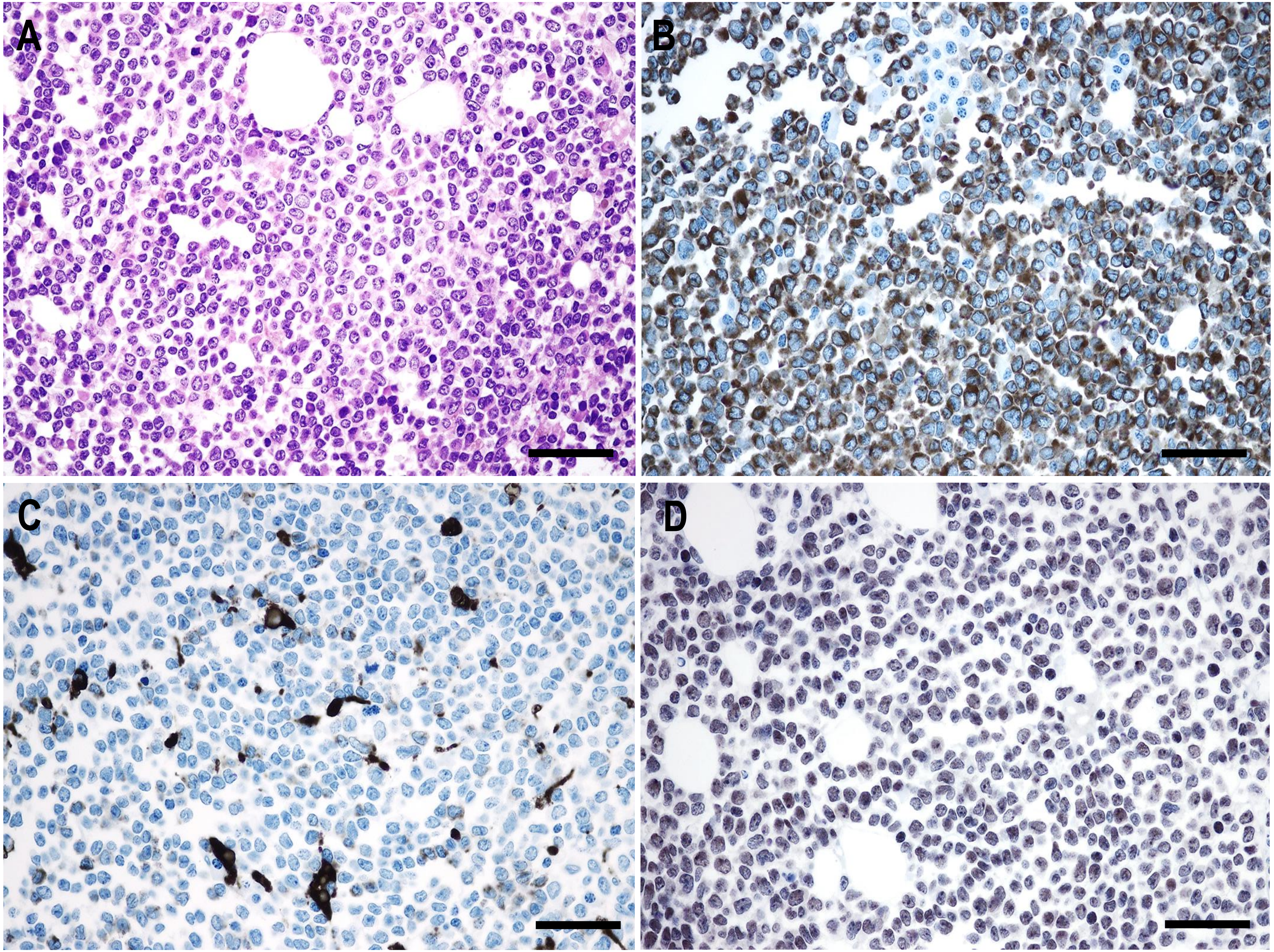


IHC of AML14.

Figure S11.

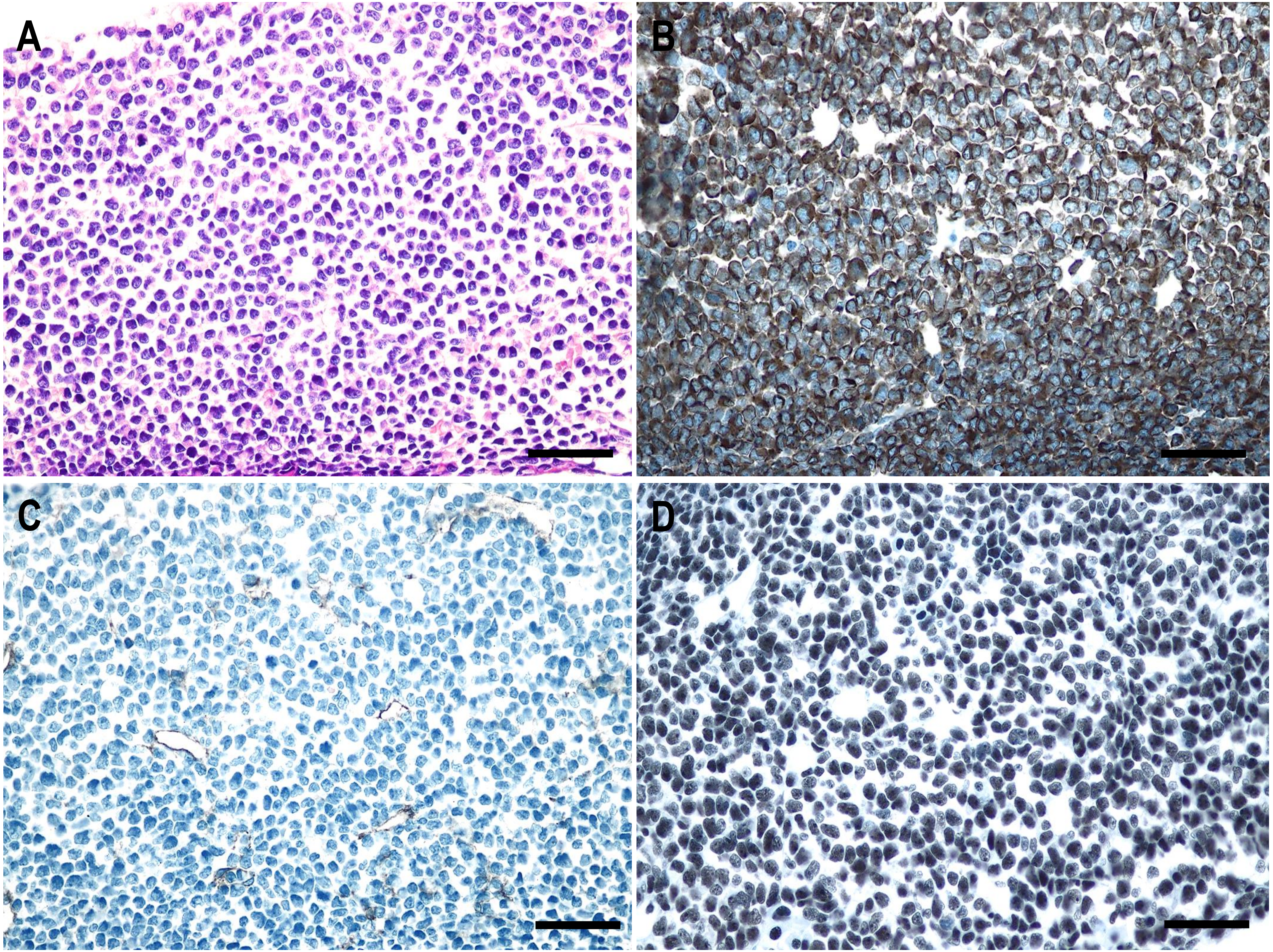


IHC of AML15.

Figure S12.

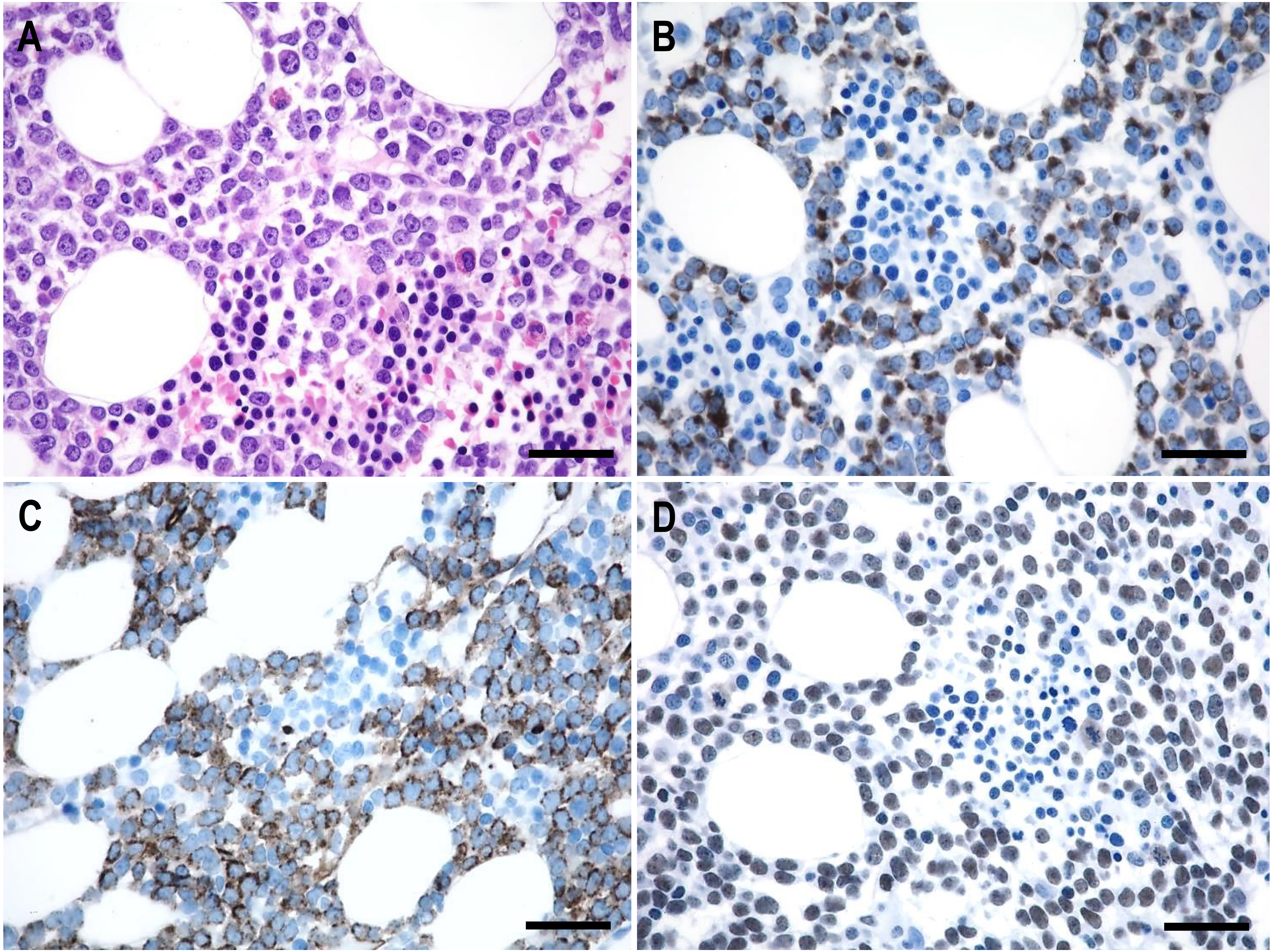


IHC of AML16.

Figure S13.

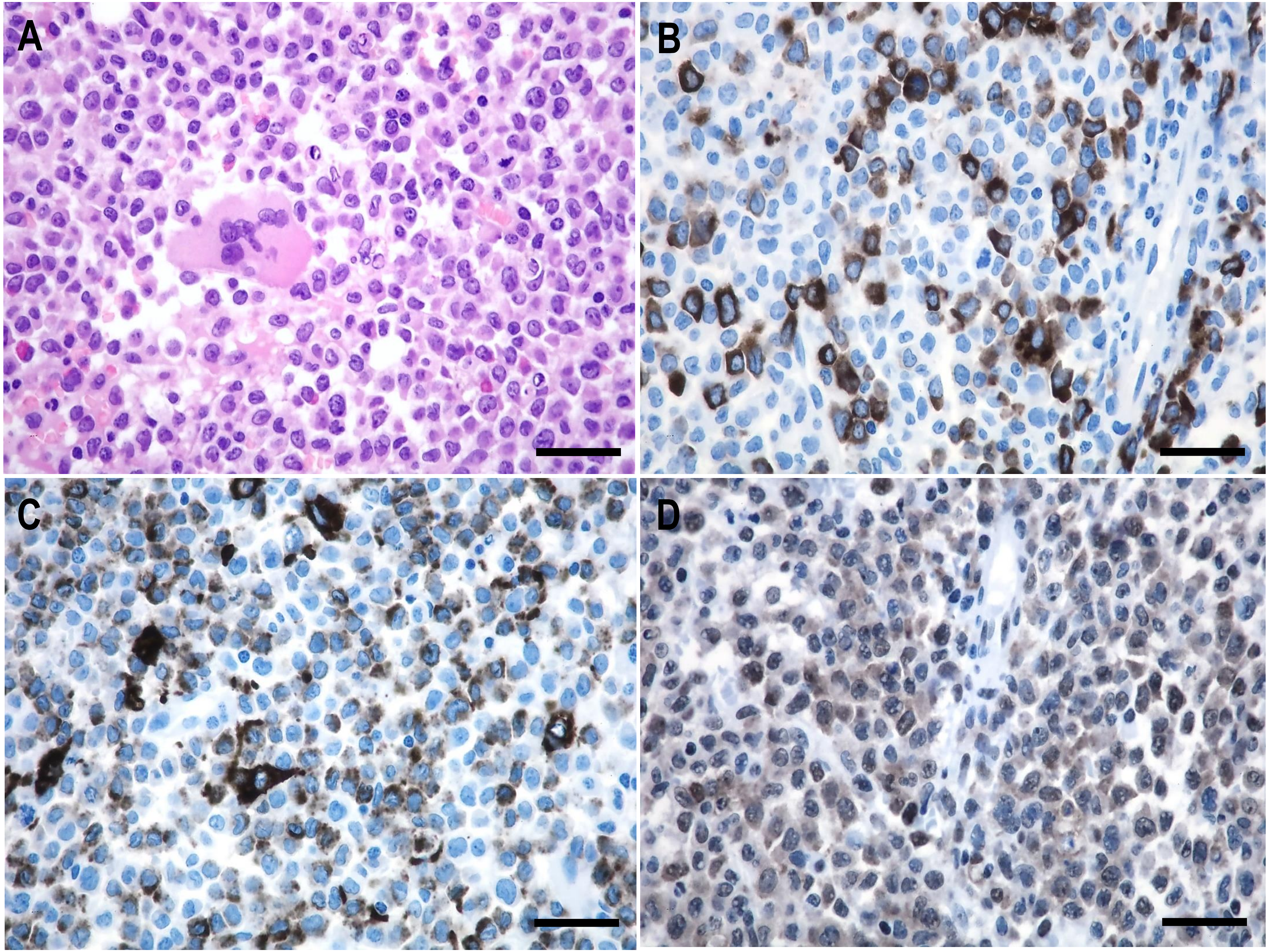


IHC of AML17.

**Supplementary Materials**

**Supplementary Materials and Methods**

## 1. Bone marrow tissue samples and immunohistochemistry

The IHC was performed as described previously with some modifications (1). We retrieved routinely stained sections of selected cases from the Laboratory of Tumor Pathology, Szeged, Hungary (except for one). One case IHC was performed at the Department of Pathology and Experimental Cancer Research, Semmelweis University, Budapest. The bone marrow trephine biopsy samples were fixed in Schaffer's fixative, decalcified using a 12.5% EDTA solution (pH 7.0), and embedded in paraffin. Immunohistochemical staining was carried out on 2-µm-thick paraffin sections following antigen retrieval with wet heat in a household electronic pressure cooker. After blocking proteins (RE7102, Leica/Novocastra, Deer Park, IL, USA), the sections were incubated with primary antibodies (**Table 1.**) at room temperature for 60 min. Detection was carried out using the Novolink Polymer Detection System (Leica/Novocastra). The immunohistochemical staining process was performed on a 4-channel TECAN Freedom Evo liquid handling platform. The IHC pathological diagnosis of the cases are shown in **Supplementary Figure 1-13**. The antibodies used for IHC analysis are listed in Supplementary **Table 1**.

**Supplementary Table 1.** The list of the antibodies used for IHC.

| **Antibody/Clone** | **Origin** | **Source** |
|---|---|---|
| CD14/5A3B11B5 | Mouse | Santa Cruz |
| CD117/PD-00-24 | Rabbit | Thermo Fisher Sc./Invitrogen |
| CD34/QBEnd/10 | Mouse | Leica/Novocastra |
| CD68/514H12 | Mouse | Leica/Novocastra |
| Ki-67/SP6 | Rabbit | Thermo Fisher Sc/Novocastra |
| Myeloperoxidase/polyclonal | Rabbit | Agilent/DAKO |
| NPM1/NA24 | Mouse | Thermo Fisher Sc. /Invitrogen |
| CD99/12E7 | Mouse | Agilent/DAKO |
| Lysosyme/EP134 | Rabbit | Sigma-Aldrich/Cell Marque |
| p53/DO-7 | Mouse | Agilent/DAKO |

## 2. Measurement of soluble proteins by Luminex MAGPIX

Plasma proteins were measured using Luminex MAGPIX as described previously by our group with minor modifications (2-4). The plasma fractions were purified using Leucosep tubes (Greiner Bio-One) and stored at -80°C in aliquots before performing the assay. The 31-Plex Human Immuno-Oncology Checkpoint Protein Panel 2 (Merck, Cat. number: HCKP2-11K) was used. The concentration of the following soluble proteins was determined using the Luminex xMAP (MAGPIX) technology according to the manufacturer's instructions: CD40 Ligand, 4-1BBL/TNFSF9, Arginase-1, B7-H2/ICOSL, B7-H3/CD276, 5'-NT/CD73, B7-H4/VTCN1, APRIL/TNFSF13, B7-H5/VISTA, CD25/IL-2Rα, B7-H6, CD137/4-1BB, Granzyme B, CD226/DNAM-1, CD30/TNFRSF8, E-cadherin, FGL1/Hepassocin, Galectin-1 (GAL1), Galectin-3, Granulysin, IDO1, MICA, MICB, Nectin-2, BAFF/Blys, Nectin-4, OX40/CD134, PVR/CD155,

Siglec-7, Siglec-9, Perforin. All samples were thawed and tested in a blind manner. Following the instructions of the kit, after washing the plate, 25 µl of standard was added to standard wells, 25 µl of assay buffer was added to the sample wells, 25 µl of matrix solution to standard wells, 25 µl of undiluted sample was added to sample wells, then 25 µL of the Mixed or Premixed Beads to each well were added. After overnight incubation and washing, biotinylated detection antibody mixture was then added to the plate and left for 1 h without washing. Following incubation, streptavidin-PE was measured to the plate. After the final washing step, 100 µl of drive fluid was added to each well, and the plate was incubated for an additional 5 min. on a plate shaker before being read on the Luminex MAGPIX instrument. Data acquisition was performed using Luminex xPonent 4.2 software. Five-PL regression curves were applied to plot the standard curves for all analytes using Analyst 5.1 (Merck) software, with bead median fluorescence intensity values used for calculation.

3. **Statistics of Luminex MAGPIX data**

Statistical analysis was performed using 14 HCs and 14 AML patients. Normally distributed datasets were compared using parametric, unpaired Welch test. For non-parametric analysis, the Mann-Whitney unpaired rank test was applied. Parametric RM one-way analysis of variance (ANOVA) was used to compare the AML follow-up samples (three time points) and healthy controls. For non-parametric analysis, the Friedman test was applied. All types of significance tests were corrected for multiple comparison by controlling the False Discovery Rate (FDR) using two-stage Benjamini, Krieger and Yekutieli approach with an FDR cutoff of 10%. The differences were considered significant at *p < 0.05; **< 0.01; ***< 0.001. Error bars indicate the mean ± standard deviation (SD).

**Supplementary Figure 1-13 Legends**

**Supplementary Figure 1.**

**IHC of AML4**. Morphology of the bone marrow in the case of AML4. (A) Blast cells accumulate in the central region of a bone marrow space (H&E, x400, scale bar=50 µm). These blasts, which make up almost 50% of the nucleated cells, are strongly CD34 positive (B) and show overlapping myeloperoxidase (C) and CD14 (D) positivity (B, C, and D x400, scale=50 µm).

**Supplementary Figure 2.**

**IHC of AML5**. Morphology of the bone marrow in the case of AML5. (A) Immature myeloid cells, many of them with nucleoli, almost completely replace normal bone marrow cells (H&E, x400, scale bar=50 µm). The atypical cells show myeloperoxidase negativity (B) and are diffusely positive for CD68R (C) (B and C x400, scale bar=50 µm). (D) The leukemic cells have a very high Ki-67 index (at least 90%) (x400, scale bar=50 µm).

**Supplementary Figure 3.**

**IHC of AML6.** Morphology of the bone marrow in the case AML6. (A) Blast equivalent atypical myeloid cells replace normal bone marrow cells (H&E, x400, scale bar=50 µm). The atypical cells show up to 30% myeloperoxidase positivity (B) and at least 20% CD68R positivity (B and C x400, scale bar=50 µm). (D) The leukemic cells have a high Ki-67 index (at least 70%) (x400, scale bar=50 µm).

**Supplementary Figure 4.**

**IHC of AML7.** Bone marrow histology of case AML7. (A) H&E image shows hypercellular bone marrow with immature myeloid proliferation representing close to 80% of the cellularity (H&E, x400, scale bar=60 µm) (A). The immature myelomonocytic population shows diffuse positivity with lysozyme (B) and heterogeneous expression of CD99 (C). Diffuse, strong nuclear p53 positivity is in line with myelodysplasia associated features (D), (B, C, and D x400, scale bar=60 µm).

**Supplementary Figure 5.**

**IHC of AML8.** Morphology of the bone marrow in the case AML8. (A) Atypical immature cells appear in the central region of a bone marrow space (H&E, x400, scale = 50 um). (B) Over 40% of the nucleated cells are CD34-positive blasts forming central clusters (x400, scale = 50 um). The blasts show partially overlapping expression of myeloperoxidase (C) and CD68R (D) (C and D x400, scale = 50 µm).

**Supplementary Figure 6.**

**IHC of AML9.** Morphology of the bone marrow in the case of AML9. (A) Atypical immature cells largely replace normal bone marrow tissue (H&E, x400, scale bar=50 µm). These cells are myeloperoxidase (B) and CD34 (C) positive, and represent myeloid blasts (B and C x400, scale bar=50 µm). (D) Over 50% of these blasts show CD68R positivity with varying intensity (x400, scale bar=50 µm).

**Supplementary Figure 7.**

**IHC of AML10.** Morphology of the bone marrow in the case AML10. (A) Atypical immature cells overgrowth the normal hemopoietic cells (H&E, x400, scale bar=50 µm). These cells show diffuse myeloperoxidase (B) and at least 60% CD68R (C) positivity (B and C x400, scale bar=50 µm). (D) Cytoplasmic NPM1 expression in atypical cells indicates a mutation (x400, scale bar=50 µm).

**IHC of AML11** was not performed. The isolation of bone marrow sample was not possible because of the condition associated with obesity of the patient.

**Supplementary Figure 8.**

**IHC of AML12.** Morphology of the bone marrow in the case AML12. (A) More than 80% of the cells in the bone marrow are blasts (H&E, x400, scale bar=50 µm). The blasts are CD34-positive (B), CD33-positive (C), and myeloperoxidase-negative (D) (B, C, and D x400, scale bar=50 µm).

**Supplementary Figure 9.**

**IHC of AML13.** Morphology of the bone marrow in the case AML13. (A) The bone marrow shows tricellular dysplasia (H&E, x400, scale bar=50 µm). (B) Dysplastic myelopoietic cells show varying myeloperoxidase positivity (x400, scale bar=50 µm). The bone marrow tissue contains up to 40% blasts with CD34 (C) and CD117 (D) positivity (C and D x400, scale bar=50 µm).

**Supplementary Figure 10.**

**IHC of AML14.** Morphology of the bone marrow in the case AML14. (A) Atypical myelomonocytic cells with mostly blast-equivalent morphology replace normal bone marrow cells (H&E, x400, scale bar=50 µm). The atypical cells show diffuse myeloperoxidase (B) and partial CD68R (C) positivity (B and C x400, scale bar=50 µm). (D) NPM1 shows uniform nuclear staining representing normal expression. Nucleoli in blast-equivalent cells are highlighted (x400, scale bar=50 µm).

**Supplementary Figure 11.**

**IHC of AML15.** Morphology of the bone marrow in the case AML15. (A) The bone marrow shows a diffuse infiltrate of atypical immature cells (H&E, x400, scale bar=50 µm). This infiltrate shows homogeneous myeloperoxidase positivity (B) and is negative for CD34 (B and C x400, scale=50 µm). (D) NPM1 expression shows normal nuclear localization. Nucleoli are visible in the leukemic cells (x400, scale = 50 µm).

**Supplementary Figure 12.**

**IHC of AML16.** Morphology of the bone marrow morphology in the case AML16. (A) Blast cells completely replace the myelopoietic compartment (H&E, x400, scale bar=50 µm). The blasts are myeloperoxidase (B) and CD34 (C) positive (B and C x400, scale bar=50 µm). (D) Blasts nuclear NPM1 expression without mutation indicating cytoplasmic staining (x400, scale bar=50 µm).

**Supplementary Figure 13.**

**IHC of AML17.** Morphology of the bone marrow in the case AML17. (A) Accumulation of atypical myelomonocytic cells with mostly blast-equivalent morphology (H&E, x400, scale bar=50 µm). These cells show partial myeloperoxidase (B) and diffuse CD68R (C) positivity (B and C x400, scale bar=50 µm). (D) NPM1 reveals mutation-defining cytoplasmic staining (x400, scale bar=50 µm).

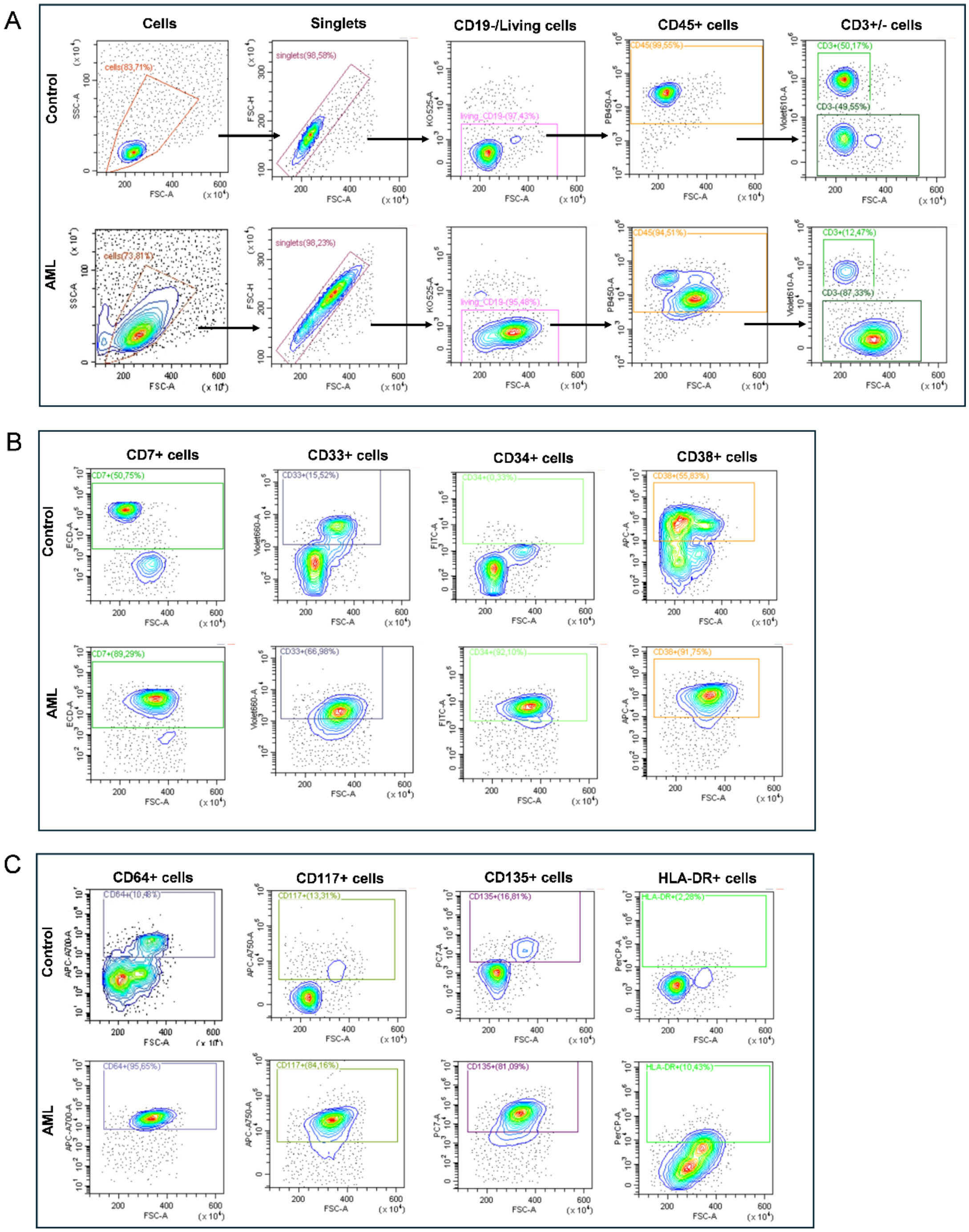


**Supplementary Figure 14.** Representative dot plots of the FACS measurement illustrating the gating strategy in CytExpert software in AML patients and healthy controls. (A) Gating strategy to define cells excluding debries, gate on single cells excluding aggregates, gate on live cells and CD19 negative cells excluding dead cells and B-cells (using the same channel), gate on CD45+ bright and dim cells to gate haematopoietic cells, gate on CD3- cells to exclude T-cells. (B) Manual gating to define CD7+, CD33+, CD34+, and CD38+ cells within the CD3-/CD19- living single cell compartment. (C) Manual gating to define CD64+, CD117+, CD135+, and HLA-DR+ cells within the CD3-/CD19- living single cell compartment.

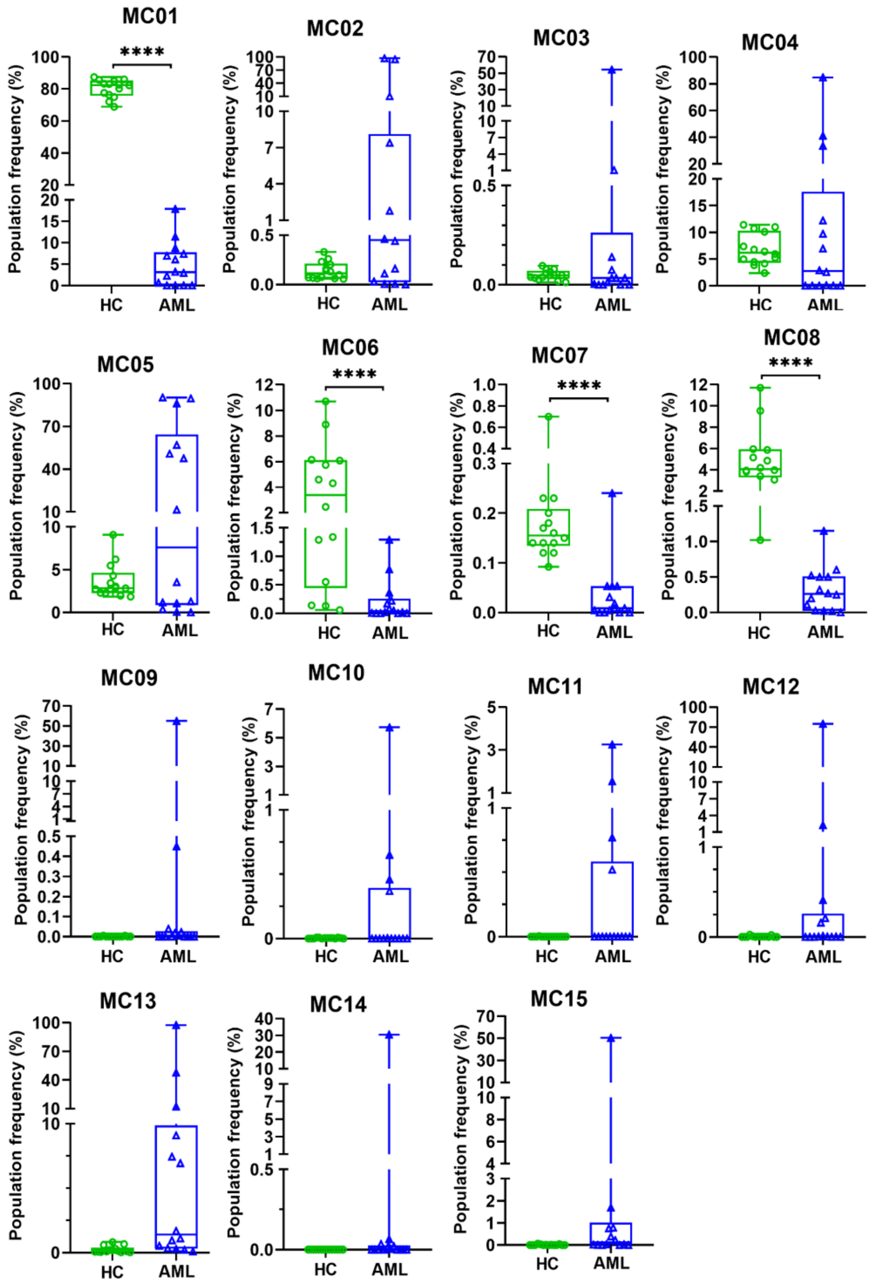


**Supplementary Figure 15.**

**Supplementary Figure 15.** The population frequency of the metaclusters in AML versus the healthy controls. The MC were determined by the FlowSOM algorithm in FlowJo software. Differences are considered significant at *p < 0.05; **< 0.01; ***< 0.001. The option for Box and whiskers graphs were set in GraphPad Prism ‘min to max, show all points’. This method plots whiskers down to the minimum and up to the maximum value, but also plots each individual value as a point superimposed on the graph. The median values are also shown within the box by an equatorial line. The boxes are extended from the 25% percentile up to 75% percentile.

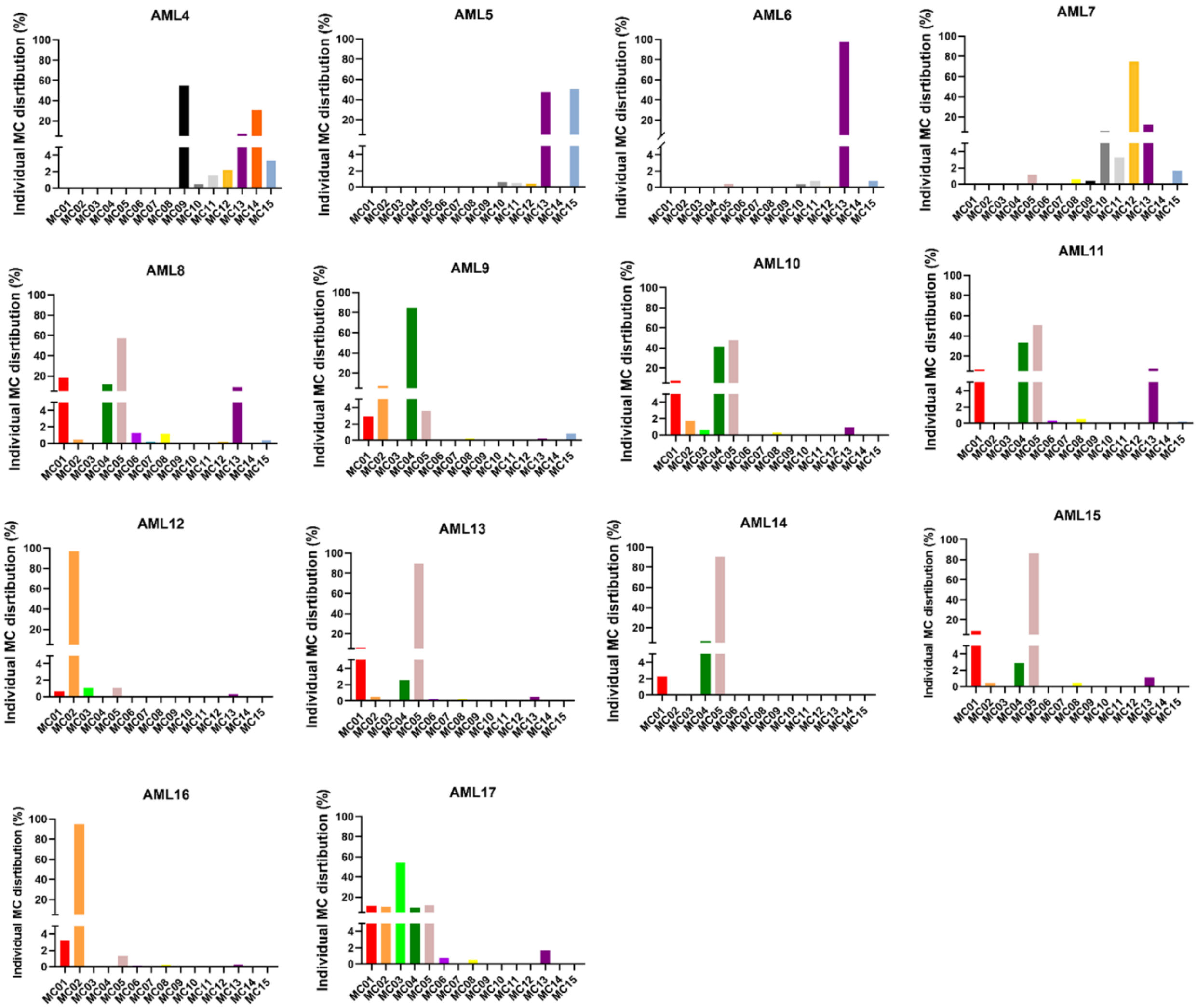


**Supplementary Figure 16.** The ratios of metacluster distributions (MC1-MC15) in the cases investigated AML (n=14). The MC were determined by the FlowSOM algorithm in FlowJo software.

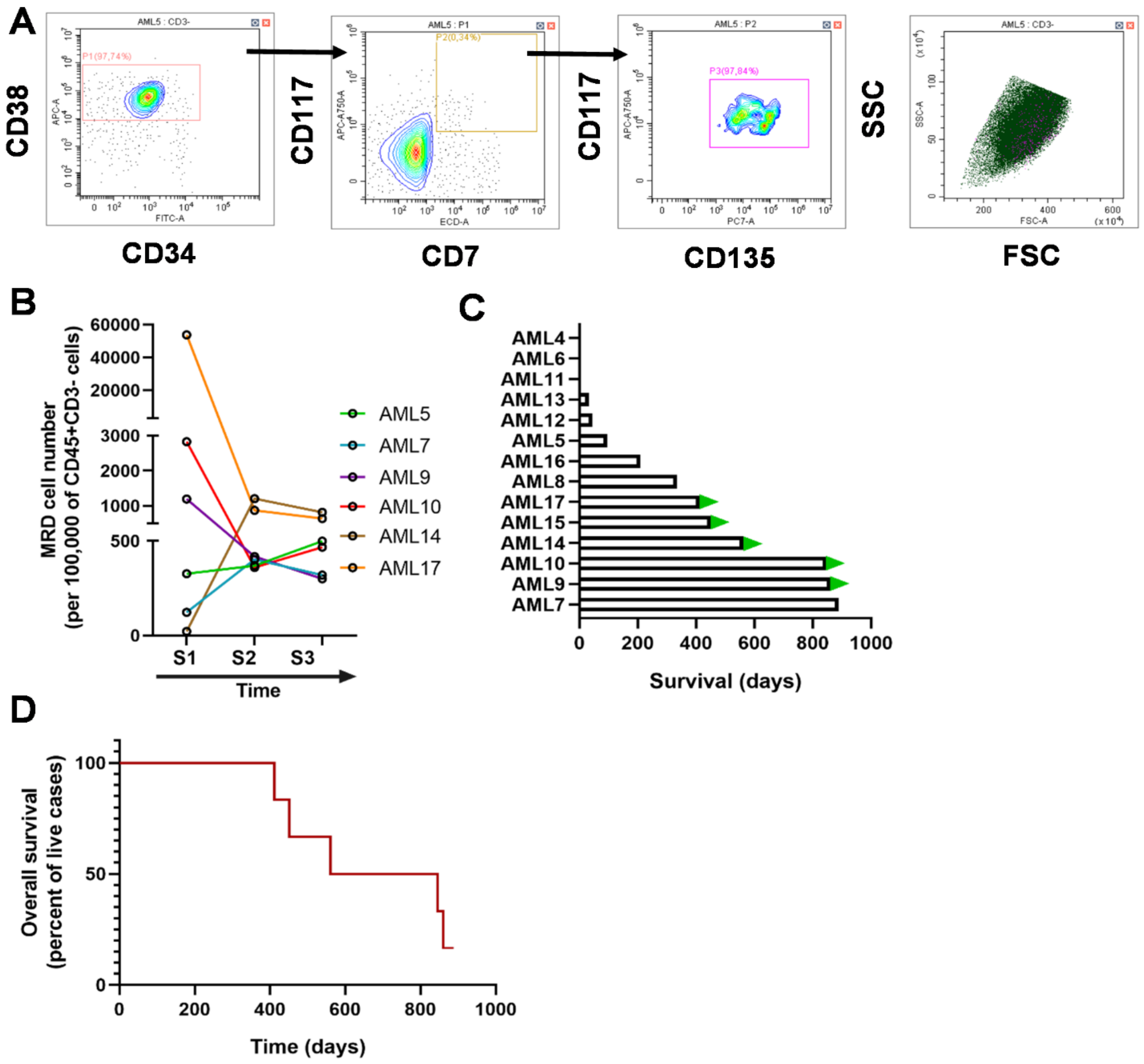


**Supplementary Figure 17.** Detection of minimal residual disease (MRD) by flow cytometry. (A) Gating strategy to detect MRD in CytExpert software. Within the CD19- living singlets, CD45+, CD3- (S. Fig. 14A), CD34-/CD38+ and the CD34+/CD38+ cells were gated (P1), then within the P1 gate the CD117+/CD7+ cells were gated as P2, then within the P2, the CD117+/CD135+ cells were gated as MRD population (P3). The identified cells (CD19-, CD45+, CD3-, CD38+/CD34±, CD7+/CD117, CD117+/CD135+) in the MRD gate are visualized on the SSC-FSC dot plots using pink color. (B) The MRD cell number was normalized to 100,000 CD45+CD3- living singlets per sample. (C) The individual survival of the AML patients studied (n=14). Green arrows represent the living subjects at the time of publication, (D) The overall survival of the studied AML cohort (n=5 living of 14 cases).

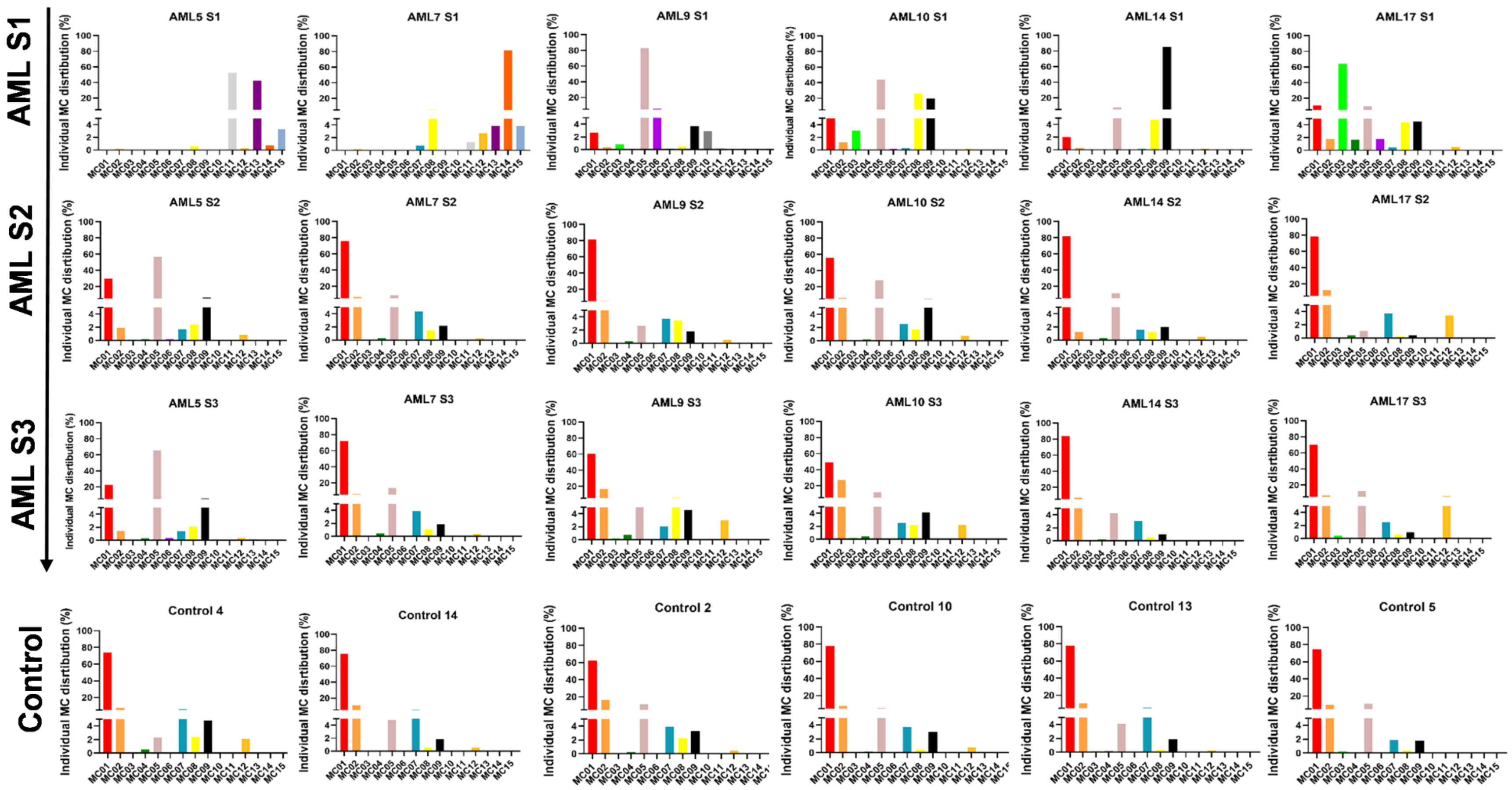


**Supplementary Figure 18.** The changes of the MC (MC1-MC15) frequencies during the cours of the therapy from time0 (S1) to the second (S2) and third (S3) sampling. The immunophenotype changed closer in similarity to controls (lower raw) in terms of the MC distribution.

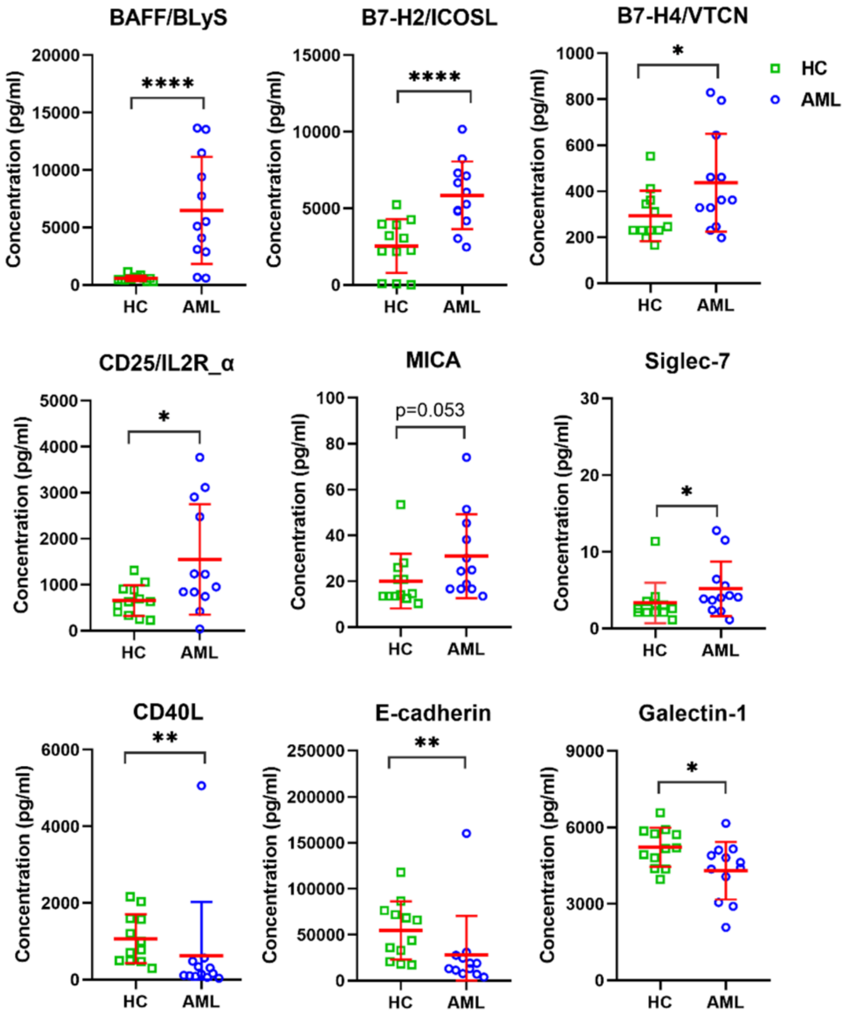


**Supplementary Figure 19. The concentration of the soluble mediators in the plasma of peripheral blood measured by the Luminex MAGPIX technology.** The 31-Plex Human Immuno-Oncology Checkpoint Protein Panel was assayed from the plasma of the patients and healthy controls (n=14/group). Only significant differences are demonstrated. Differences are considered significant at *p < 0.05; **< 0.01; ***< 0.001. Red error bars specify mean ± SD.

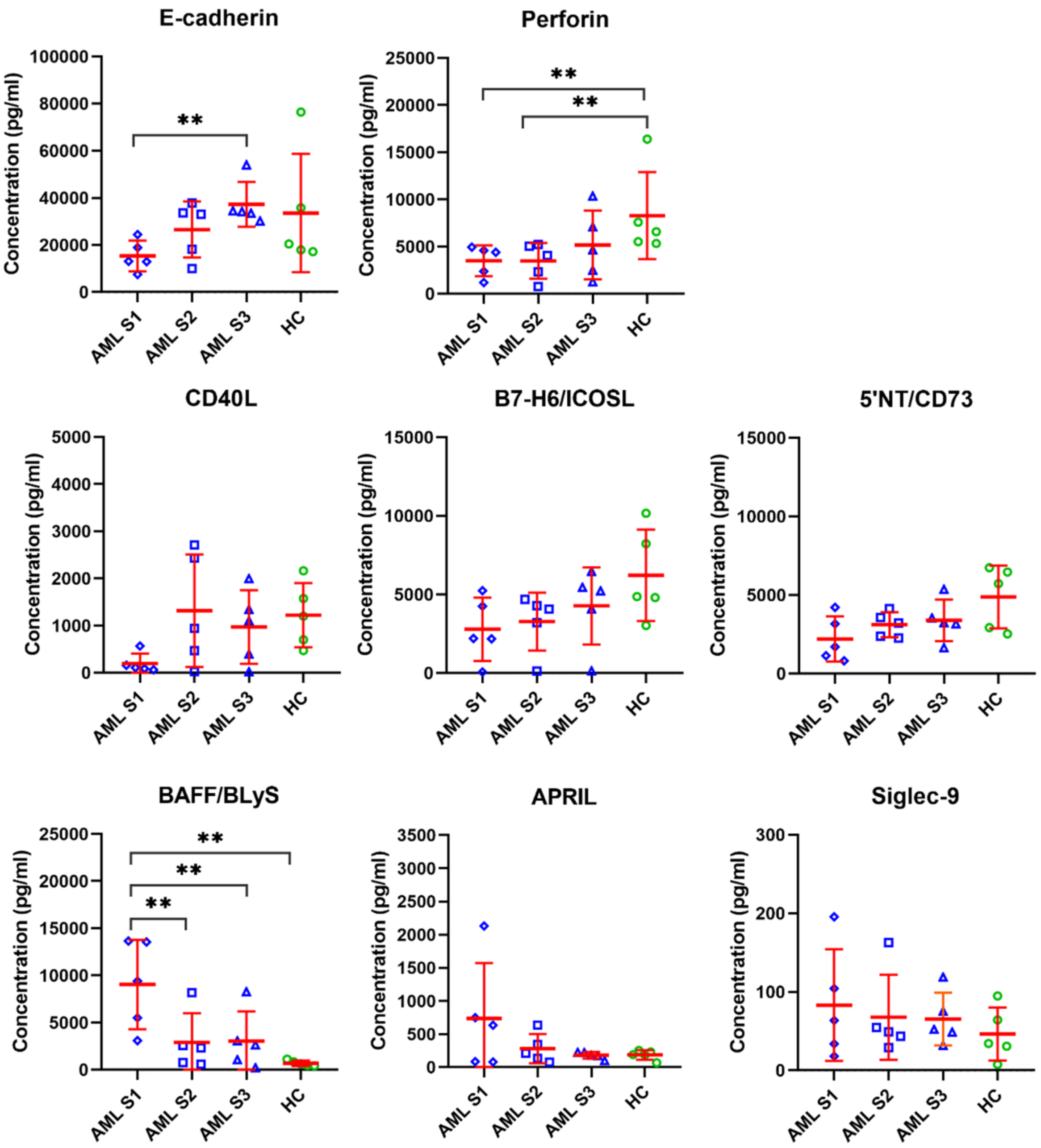


**Supplementary Figure 20. The concentration of the soluble mediators in the plasma of peripheral blood of the patients during the follow-up measured by the Luminex MAGPIX technology.** The 31-Plex Human Immuno-Oncology Checkpoint Protein Panel was assayed from the plasma of the patients and healthy controls. Differences are considered significant at *p < 0.05; **< 0.01; ***< 0.001. Red error bars specify mean ± SD.

1. Krenacs L, Krenacs D, Borbenyi Z, Toth E, Nagy A, Piukovics K, et al. Comparison of Follicular Helper T-Cell Markers with the Expression of the Follicular Homing Marker CXCR5 in Peripheral T-Cell Lymphomas-A Reappraisal of Follicular Helper T-Cell Lymphomas. *Int J Mol Sci* (2023) 25(1). Epub 2024/01/11. doi: 10.3390/ijms25010428. PubMed PMID: 38203606; PubMed Central PMCID: PMCPMC10778845.
2. Balog JA, Kemeny A, Puskas LG, Burcsar S, Balog A, Szebeni GJ. Investigation of Newly Diagnosed Drug-Naive Patients with Systemic Autoimmune Diseases Revealed the Cleaved Peptide Tyrosine Tyrosine (PYY 3-36) as a Specific Plasma Biomarker of Rheumatoid Arthritis. *Mediators Inflamm* (2021) 2021:5523582. Epub 2021/07/10. doi: 10.1155/2021/5523582. PubMed PMID: 34239365; PubMed Central PMCID: PMCPMC8240466 publication of this paper.
3. Toth ME, Dukay B, Peter M, Balogh G, Szucs G, Zvara A, et al. Male and Female Animals Respond Differently to High-Fat Diet and Regular Exercise Training in a Mouse Model of Hyperlipidemia. *Int J Mol Sci* (2021) 22(8). Epub 2021/05/01. doi: 10.3390/ijms22084198. PubMed PMID: 33919597; PubMed Central PMCID: PMCPMC8073713.
4. Gemes N, Balog JA, Neuperger P, Schlegl E, Barta I, Fillinger J, et al. Single-cell immunophenotyping revealed the association of CD4+ central and CD4+ effector memory T cells linking exacerbating chronic obstructive pulmonary disease and NSCLC. *Front Immunol* (2023) 14:1297577. Epub 2024/01/08. doi: 10.3389/fimmu.2023.1297577. PubMed PMID: 38187374; PubMed Central PMCID: PMCPMC10770259.
